# Progress in advanced nanotherapeutics for enhanced photodynamic immunotherapy of tumor

**DOI:** 10.7150/thno.73566

**Published:** 2022-07-04

**Authors:** Xiao Wei, Mingzhu Song, Guirong Jiang, Min Liang, Chunlan Chen, Zhiyong Yang, Liang Zou

**Affiliations:** 1 School of Preclinical Medicine, Chengdu University, Chengdu 610106, P. R. China.; 2 School of Food and Bioengineering, Chengdu University, Chengdu 610106, P. R. China.; 3 Key Disciplines of Clinical Pharmacy, Affiliated Hospital and Clinical Medical College of Chengdu University, Chengdu 610081, P. R. China.; 4 Department of Thoracic and Cardiac Surgery, Affiliated Hospital of Chengdu University, Chengdu 610081, P. R. China.

**Keywords:** photodynamic therapy, immunogenic cell death, nanotherapeutics, tumor immunotherapy

## Abstract

Clinically, the conventional treatments of cancer are still often accompanied by tumor recurrence, metastasis and other poor prognosis. Nowadays, more attention has been paid to photodynamic therapy (PDT), which is regarded as an adjuvant antineoplastic strategy with superiorities in great spatiotemporal selectivity and minimal invasiveness. In addition to eliminating tumor cells via reactive oxygen species (ROS), more meaningfully, this phototherapy can trigger immunogenic cell death (ICD) that plays a vital role in photodynamic immunotherapy (PDIT). ICD-based PDIT holds some immunotherapeutic potential due to further enhanced antitumor efficacy by utilizing various combined therapies to increase ICD levels. To help the PDIT-related drugs improve pharmacokinetic properties, bioavailability and system toxicity, multifunctional nanocarriers can be reasonably designed for enhanced PDIT. In further consideration of severe hypoxia, low immunity and immune checkpoints in tumor microenvironment (TME), advanced nanotherapeutics-mediated PDIT has been extensively studied for boosting antitumor immunity by oxygen-augment, ICD-boosting, adjuvant stimulation and combined checkpoints blockade. Herein, this review will summarize different categories of nanocarriers consisting of their material type, targeting and stimuli-responsiveness. Moreover, we will focus on the latest progress of various strategies to enhance the antitumor immune effect for PDIT and elucidate their corresponding immune-activation mechanisms. Nevertheless, there are several thorny challenges in PDIT, including limited light penetration, tumor hypoxia, immune escape and the development of novel small-molecule compounds that replace immune checkpoint inhibitors (ICIs) for easy integration into nanosystems. It is hoped that these issues raised will be helpful to the preclinical study of nanotherapeutics-based PDIT, thus accelerating the transformation of PDIT to clinical practice.

## Introduction

As a global malignant disease, cancer usually shows a very high mortality rate. The main clinical treatments for cancer are still surgery, chemotherapy and radiotherapy nowadays 1. Unfortunately, high recurrence, high metastasis and other poor prognosis in cancer patients can't be effectively avoided only by employing the conventional clinical medication. To address these problems, some antitumor adjuvant therapies like photodynamic therapy (PDT) have been gradually developed, which refers to the conversion of oxygen (O_2_) to cytotoxic ROS via photosensitizers (PSs) under light, and directly triggering the intrinsic mitochondrial oxidative damage-related apoptotic pathways, thereby achieving the goal of killing tumor cells 2, 3. The dominant features of PDT present great spatiotemporal selectivity and minimal invasiveness 4. In addition to destroying tumor cells by ROS, PDT can even induce immunogenic cell death (ICD), which is accompanied by the generation and release of damage-associated molecular patterns (DAMPs) including calreticulin (CRT), high mobility group box 1 (HMGB1) and adenosine triphosphate (ATP), which can be recognized by a variety of immune cells, and thus provoking the specific antitumor immune response 5, 6. Numerous studies have demonstrated that ICD-based immunotherapeutic strategies hold great potential in cancer therapy 7, 8. Thus more and more attention has also been paid to ICD-focused PDIT that owns an enhanced suppressive effect on certain tumors.

Visible light can be applied to penetrate tissues and trigger conventional PSs to generate ROS for PDT. Whereas, owing to the insufficient penetration of visible light, deep tumors become difficult to eradicate effectively 9. Moreover, most of the PSs such as chlorin e6 (Ce6) and protoporphyrin IX (PpIX) in the free state usually show some disadvantageous properties, including low stability, suboptimal selectivity, poor cell absorption and poor tumor retention 10, 11. Besides, the common immunomodulators like imiquimod (R837) are easy to be eliminated quickly while transported in the body. Meanwhile, as a result of the limited ability of tumor targeting and the complexity and heterogeneity of tumor microenvironment (TME), it's hard to develop the precise targeted therapeutic reagents 12. To overcome the above defects, hydrophilic and surface-modifiable nanomaterials, which can be passively accumulated in tumor sites via the enhanced permeability and retention (EPR) effect, are widely applied in tumor PDIT 13, 14. As a thriving therapeutic modality, the nanomaterials-based advanced nanotherapeutics can effectively assist drug delivery, thus improving the therapeutic effect of drugs. Specifically, the nanocarriers like liposomes, polymeric micelles or other nanomaterials can easily carry PSs (e.g., Ce6, PpIX), immune adjuvants (e.g., R837, cytosine-phosphate-guanine (CpG)), immune checkpoint inhibitors (ICIs) (e.g., small interfering RNA (siRNA), antibody), chemotherapy drugs (e.g., doxorubicin (DOX), paclitaxel), etc. to directly target tumor sites, further enhancing the stability of the delivery drugs and reducing systemic toxicity via the encapsulation and release of drugs 15, 16.

Tumor cells can be effectively eliminated by advanced nanotherapeutics-mediated PDIT. To be specific, tumor ICD can be induced after realizing PDT effect, to further promote the maturation of antigen-presenting cells (APCs) like dendritic cells (DCs) that can activate effector T cells, and thus advancing the immunosuppressive effect on tumor cells. However, there are some constraints such as severe hypoxia, poor ICD and immune escape that greatly suppress the antitumor efficacy during ICD-based PDIT. Among them, high hypoxia in TME results in limited efficacy of O_2_-dependent PDT 17. Increasing evidence has indicated that, catalase (CAT) and manganese dioxide (MnO_2_) can catalyze endogenous hydrogen peroxide (H_2_O_2_) to produce O_2_ 18, 19. So it is usually considered to alleviate tumor hypoxia by using nanocarriers loading CAT, MnO_2_ or other O_2_ catalysts in PDIT. Additionally, direct delivery of O_2_ through oxygen carriers such as perfluorocarbons (PFCs) and hemoglobin (Hb) is also a feasible way to remodel the tumor hypoxic microenvironment. Furthermore, the low-level ICD induced by common PDT strategies has become a critical concern in PDIT 20. So far, the combination strategies like PDT synergized with chemotherapy or photothermal therapy (PTT) have been developed for increased tumor ICD 21, whose characteristics are to expedite antigen presentation via CRT exposure, HMGB1 release and ATP secretion. Similarly, some adjuvants like CpG can be easily integrated into nanoplatforms, which accelerate antigen presentation by stimulating toll-like receptors (TLRs) and further provoke effector T cells, thus enhancing specific antitumor immunity. Besides, immune escape of tumor cells becomes the one of essential features of tumor progression 22. On account of the lack of costimulatory molecules that are able to activate T cells in immunosuppressive TME, fewer effector T cells can be generated and infiltrated into the tumor region, implying that malignant tumor cells can easily elude immune surveillance 23, 24. As a dominant driver of tumor immune escape, immune checkpoints, such as programmed cell death protein 1 (PD-1)/programmed death-ligand 1 (PD-L1), cytotoxic T-lymphocyte-associated protein 4 (CTLA4) and indoleamine 2,3-dioxygenase (IDO), have long become a topic of great interest in biomedical field 25-27. Generally, activated T cells can be notably inhibited by those immune checkpoints after antigen recognition 28, which leads to an inadequate antitumor immunity. In the past decade, the clinical application of immune checkpoint inhibitors (ICIs) has greatly promoted the development of cancer immunotherapy 29-31. On the whole, the combination of ICD-based PDIT with immune checkpoint blockade (ICB) can bring about a superior tumor-suppressive potency by successfully inhibiting tumor immune escape.

**Scheme 1:**
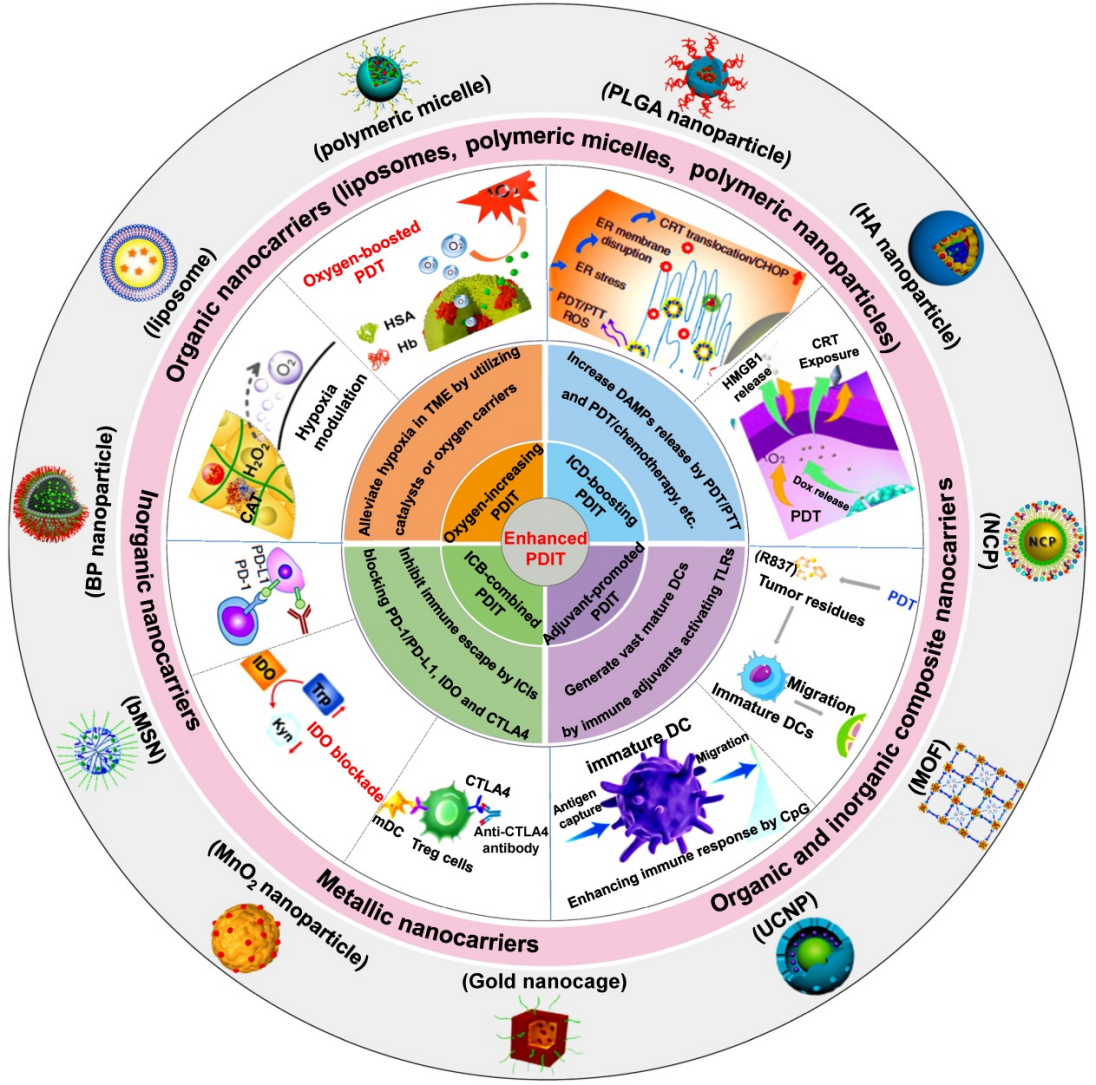
Schematic illustration of various advanced nanotherapeutics for enhanced PDIT. Partial images were adapted with permission from 32 copyright 2019, 33 copyright 2019, 34 copyright 2016, 35 copyright 2019 WILEY-VCH Verlag GmbH & Co. KGaA, Weinheim; 36 copyright 2018, 37 copyright 2019, 9 copyright 2017, 38 copyright 2019, 39 copyright 2016, 40 copyright 2015, 41 copyright 2020, 42 copyright 2019 American Chemical Society; 43 copyright 2019, 44 copyright 2016 Springer Nature Limited; 45 copyright 2021, 46 copyright 2021 Wiley‐VCH GmbH; 47 copyright 2019, 48 copyright 2018 Ivyspring International Publisher; 49, copyright 2020 Springer Nature Switzerland AG; 50, copyright 2018 Elsevier Ltd.

In this review, we first introduce the material property of various nanocarriers containing organic nanocarriers, inorganic nanocarriers, metallic nanocarriers, and organic and inorganic composite nanocarriers, and concretely describe their functional diversities that include specific cell targeting and stimuli-responsiveness. Then, we discuss in depth the mechanisms and applications of several strategies associated with nanotherapeutics-mediated PDIT, which contain “Oxygen-increasing PDIT”, “ICD-boosting PDIT”, “Adjuvant-promoted PDIT” and “ICB-combined PDIT” **(Scheme 1)**. Last, we summarize the current challenges and development prospects of PDIT, and put forward possible solutions to its existing defects and exposed problems. We hope to expand the advantages of advanced nanotherapeutics-based PDIT, thus shortening the transformation of PDIT from laboratory research to clinical trials.

### Reasonable design of nanocarriers for PDIT

Nanocarriers-based PDIT is increasingly appreciated as an alternative antitumor strategy. Generally, nanometer size is regarded as a typical physical feature in nanosystem. Notably, the size of nanocarriers can usually be controlled to be relatively big to avoid invading capillaries, while also being small enough to avoid phagocytosis by the macrophages of the reticuloendothelial system 51. Hence, suitable particle size of nanocarriers should be designed for PDIT under certain conditions to achieve prolonged circulation and selective extravasation 52. Except for the particle size, the shape, charge and composition of functional nanocarriers also play a vital role in helping the payloads improve safety, pharmacokinetic properties and bioavailability, while determining the tissue distribution and cell internalization of drugs 53. In addition, targeted nanocarriers based on different targeting molecules can achieve accurate localization and accumulation in tumor sites via active targeting or passive targeting like EPR. Particularly, active targeting can enhance therapeutic efficacy by increasing cell specificity and uptake 54. Also, stimulus-responsive nanocarriers are able to control the cargoes release to specific sites under different external or internal stimuli, thus increasing the concentration of drug in tumor region. Collectively, reasonable design of various targeted and responsive nanocarriers with different sizes, shapes, charges and compositions is essential for antitumor accuracy and effectiveness of PDIT. Next, here we summarize the structural characteristics of various nanoplatforms for delivering cargoes in PDIT, and their corresponding functionalities such as targeting and stimulus response **(Table 1)**.

### Different types of nanocarriers for delivering drugs

Multiple types of nanocarriers are capable of being utilized for delivering PDIT-related antitumor drugs. In the section, we mainly discuss four types of nanocarriers, including organic nanocarriers, inorganic nanocarriers, metallic nanocarriers, and organic and inorganic composite nanocarriers.

#### Organic nanocarriers

#### Liposomes

Liposomes, the spherical vesicles composed of cholesterol, phosphatidylcholine and phosphatidylethanolamine, have a hydrophilic core and double-shell with good biocompatibility 63, 64. Although conventional liposomal nanocarriers have some disadvantages such as the poor stability and rapid removal from blood circulation 65, liposomes can still be widely applied to encapsulate and deliver a variety of drug molecules, such as peptides, siRNA, chemotherapy drugs, etc., thus effectively reducing the toxicity of drugs, improving their pharmacokinetic properties, and enhancing the efficacy of tumor therapy 66. In general, liposomes can integrate therapeutic reagents through active extrusion or passive diffusion. However, since different drugs have diverse pharmacokinetic characteristics, there are some differences in the form of packages between these drugs. Specifically, the hydrophilic drugs can enter the inner core of liposomes by passive diffusion, and the lipophilic drugs can be loaded into lipid bilayer resulted from hydrophobic interactions. Of course, liposomes can also synchronously encapsulate both hydrophilic and hydrophobic drugs to fulfill the co-delivery. Besides, nucleic acid-based drugs with negative charge can be carried and delivered by cationic charge adsorption effect on the surface of liposomes 67, 68. Ding et al. used a liposome loaded with Ce6 and phosphoinositide 3-kinase gamma (PI3Kγ) inhibitor IPI-549 through hydrophobic interaction, which was capable of targeting myeloid-derived suppressive cells (MDSCs) in tumor immune microenvironment (TIME) and reversing the immunosuppressive state, thus enhancing PDIT efficacy to eradicate colon cancer 55. Additionally, Kim et al. reported a liposome that encapsulated gemcitabine (GEM) in a hydrophilic core through hydrophilic interaction, which was beneficial to the treatment of biliary duct cancer by employing the therapeutic pattern of PDIT 56. Similarly, Wang et al. also reported a redox-activatable liposome constructed by phospholipid-porphyrin conjugates, where IDO inhibitor NLG-8189 was embedded via hydrophilic forces, leading to the obvious augment of tumor ICD level, thus boosting the therapeutic effect of systemic anti-tumor immunity 38.

##### Polymeric micelles

Typically, polymeric micelles with spherical structure can be formed spontaneously while amphiphilic block copolymers are put into an aqueous solution, which consist of a hydrophobic inner core and a hydrophilic outer shell 69. Unfortunately, polymeric micelles are less stable in biological fluids and have more complex properties 70, but there are still numerous advantages in drug delivery for tumor therapy. Specifically, the core of polymeric micelles can be made up of hydrophobic polymer segments, in which liposoluble drugs such as debydrochlorination DOX and paclitaxel can be effectively wrapped by hydrophobic effect 71. Additionally, the hydrophilic components of block copolymers constitute the hydrophilic shell of micelles to ensure the high solubility of polymeric micelles in blood, thus facilitating the targeted delivery of drugs 72. At present, it is common to design different types of polymeric micelles to deliver chemotherapeutic drugs for anti-tumor PDIT. For instance, Wang et al. designed a polymeric micelle based on negatively charged siRNA that could be adsorbed onto the surface of the micelle shell via electrostatic adsorption effect 39. The polymeric micelle was unassembled under weak acid in TME, and siRNA was subsequently released to achieve PD-L1 gene knockdown (KD), resulting in enhanced immunosuppression against metastatic lung tumors. Cai et al. also used the same micelle embedding siRNA to achieve PD-L1 KD, which increased the number of CD8^+^ T cells and generated a potent antitumor immunity for PDIT 73. In addition, Peng et al. designed a micelle modified by triphenylphosphonium (TPP) groups called as TPPM, which encapsulated the Ce6 in the hydrophobic core through physical hydrophobic forces, and was loaded with TPP on the surface of the shell via covalent grafting, thus being conducive to realizing the increased PDIT after targeting the mitochondria of tumor cells 74.

##### Polymeric nanoparticles

Polymeric nanoparticles (NPs), usually possess a size of 10-500 nm and a spherical structure, can be constructed by the self-assembly of natural or synthetic copolymers such as poly(lactic-co-glycolic acid) (PLGA), polysaccharides and natural proteins. Notably, remarkable varies of achievable drug loading in polymeric NPs limit their extensive application 75. But undoubtedly, owing to favorable physicochemical features (e.g., easily controllable size and surface charge, the availability of various functional groups to conjugate cargoes) 76, polymeric NPs have become the greatly attractive nanocarriers. Generally, polymeric nanospheres or nanocapsules can be formed to wrap and deliver therapeutic drugs for cancer therapy 77. Namely, the nanosphere is composed of the polymeric matrix, where drugs can be physically retained or adsorbed by electrostatic forces; the nanocapsule is made up of a polymeric shell that wraps around an oily core, in which hydrophobic drugs can be dissolved. It should be noted that the drugs can also be adsorbed on the wall of the polymeric shell 78. For PLGA NPs, Yang et al. constructed a composite nanostructure containing the PLGA nanocompartment and ferritin (FRT) nanocompartment 79. Specifically, the IDO inhibitor NLG919 was encapsulated in the PLGA nanocompartment through hydrophobic forces, and the zinc hexadecafuoro-phthalocyanine (ZnF16Pc) as a PS was physically loaded into the FRT nanocompartment, ultimately leading to an optimal tumor suppression by enhanced PDIT. Moreover, Ou et al. reported a PLGA NP modified by GITR antibody that was a kind of tumor necrosis factor (TNF) receptor family-related proteins, which simultaneously encapsulated imatinib (IMT) into the core of the nanocarrier via hydrophobic forces and absorbed the positively charged PS IR-780 on the polymeric shell by electrostatic interaction, thus quickly damaging tumor cell membrane during PDIT 48. For polysaccharide NPs, hyaluronic acid (HA) NPs are widely applied in the relevant research of tumor PDIT. Sun et al. proposed a HA NP called as HCJSP based on HA-CD, AD-SS-JQ1 and AD-SS-PPa for pancreatic cancer therapy, in which HA-CD consisted of cyclodextrin (CD) grafting HA, and AD-SS-JQ1 and AD-SS-PPa were constituted by adamantine (AD) connecting with bromodomain and extraterminal protein 4 (BRD4) inhibitor JQ1 or pyropheophorbidea (PPa) via disulfide bonds, respectively 46. Interestingly enough, the drug-loading pattern of HCJSP was different from the conventional modes such as hydrophobic or electrostatic interaction. In this study, both CD and AD were conjugated by molecular docking and host-guest interaction for the introduction of JQ1 and PPa into the HCJSP nanosystem. For natural protein NPs, Hu et al. synthesized a polymeric NP based on polypeptides (MA-pepA-Ce6 NP) that could be cleaved by matrix metalloproteinase-2 (MMP-2), where Ce6 was covalently grafted into the hydrophobic nanocore via amido bond, and metformin (MET) was conjugated into this polypeptides-based nanoplatform via the acid-sensitive bond 58. As expected, this tactic effectively inhibited the PD-L1 ligand of tumor cells, thus enhancing the anti-breast cancer immune response of PDIT. In addition, dopamine is also applied to synthesize polymeric NPs. Wu et al. fabricated a hybrid polymer NPs consisted of polydopamine (PDA) and upconversion nanoparticle (UCNP), where Ce6 was encapsulated on the surface of mixed NPs for the combination of PDT/PTT. Specifically, the internal PDA was regarded as the photothermal core for PTT and the UCNP shell was used for PDT. Under 980 nm laser irradiation, synergistic photoimmunotherapy could achieve the augmented level of tumor ICD by exposing CRT, and further induced abundant mature DCs to activate cytotoxic T lymphocytes (CTLs), ultimately generating a potent antitumor immunity and effectively inhibiting tumor metastasis 80.

#### Inorganic nanocarriers

As a suitable inorganic nanocarrier, black phosphorus (BP) has been widely used in cancer treatment due to its advantages of high biocompatibility, good biodegradation, large surface area and negative charge 81. For example, Li et al. constructed a novel NIR/ROS-sensitive BPQDs nanovesicle (BPNV), which encapsulated CpG into the vesicular cavity for antitumor immune activation in PDIT, leading to extensive damage of tumor cells via increasing TNF-α, IL-6 and IL-12 in serum 35.

As is well-known, the graphene with a two-dimensional (2D) and honeycomb structure possesses a large surface area to easily load more PSs, targeting moieties or chemotherapy drugs 82. However, as a 0D graphene material, graphene quantum dots (GQDs/GOQDs) are highly desirable for antitumor immunity 60, which exhibit good distribution characteristics within tumor cells and show low toxicity to surrounding tissues. Also, it has been demonstrated that, the single-layer structure of graphene can increase its transparency to facilitate light penetration, and its internal π-π stacking structure can be easier to cooperate with the drug molecules, thus improving its overall solubility and stability 83. Nafiujjaman et al. employed the GQDs binding with Ce6 and HA via ester bonds for tumor PDIT, which promoted the production of large amounts of ROS, and thus triggered a strong antitumor immune response 60.

Likewise, mesoporous silica (SiO_2_) NPs, that also own a high specific surface area and pore volume, can enable the delivery of more antitumor cargoes 84. Additionally, good biocompatibility of SiO_2_ can ensure the endocytosis of more drugs 62. Xu et al. designed a biodegradable mesoporous SiO_2_ NP (bMSN) loading CpG via electrostatic absorption and Ce6 through hydrophobic interaction, which expedited the maturation of DCs in PDIT, ultimately showing strong antitumor effects on both local and distant tumors in C57BL/6 mice 42.

#### Metallic nanocarriers

As a common metal-O_2_ catalyst, MnO_2_ with large surface areas, good absorption and degradation abilities has been extensively applied in antitumor PDIT 85. Zhou et al. constructed a multifunctional anti-cancer nanoplatform, in which the mesoporous SiO_2_ shell and MnO_2_ shell were separately coated on the surface of Cu9S5 nanocrystals, and the adjuvant CpG was also loaded by electrostatic forces, contributing to producing a large number of ROS to destroy tumor cells 86.

Furthermore, although magnetic ferriferrous oxide (Fe_3_O_4_) NPs possess low toxicity, they are still greatly valuable for the delivery of anti-cancer drug. Moreover, Fe_3_O_4_ can also catalyze the decomposition of endogenous H_2_O_2_ to produce O_2_, thereby significantly improving the internal hypoxia of tumors 87. For instance, Wang et al. prepared the Janus magnetic mesoporous organosilicon NPs (M-MONs) constituted by Fe_3_O_4_ head and SiO_2_ body to load the Ce6, which showed an obvious inhibition effect on primary and distant tumors 62.

#### Organic and inorganic composite nanocarriers

As a common organic and inorganic composite nanomaterial, non-toxic and porous MOFs consist of metal ions or clusters and organic ligands, which can reduce toxicity to surrounding tissues and enhance the loading capacity 88. Lan et al. developed a novel MOFs-based PS (Fe-TBP) that was composed of Fe_3_O clusters and 5,10,15,20-tetra(*p*-benzoato)porphyrin (TBP) ligand. After the PDT effect was achieved under light, the anti-PD-L1 antibody was further administrated to realize the combined treatment of PDT and ICB, thus leading to increased CD4^+^ and CD8^+^ T cells that can specifically eliminate tumor cells. From* in vivo* outcomes, tumor elimination rate reached more than 90% in the mouse model of colorectal cancer 89.

For another typical composite material, UCNPs can be more stably transferred to aqueous solutions responsible for their oleic acid stability 90. Moreover, UCNPs can generate large amounts of oxygen radicals under NIR to disrupt the redox homeostasis within tumor cells 91. For the research on UCNPs, Xu et al. designed the UCNPs for the treatment of colorectal cancer. Namely, UCNPs were loaded with Ce6 and R837 via hydrophobic forces, which facilitated the maturation of DCs and release of related cytokines, further markedly reinforcing the efficacy of PDIT by combining PDT with CTLA4-blockade therapy 9.

Furthermore, the nanoscale coordination polymers (NCPs), composed of organic bridging linkers and metal ions, possess highly tunable constituents and structures, which can enable the combination of various therapeutic cargoes or modalities 92, 93. By this token, NCPs may be also commonly applied in the sufficient delivery of multiple antitumor reagents during PDIT. He et al. reported a NCP nanoparticle carrying chemotherapeutic cisplatin and PS pyrolipid for enhanced PDIT, which could exhibit marked therapeutic effect on head and neck cancer via the combination of PDT and chemotherapy 40.

### Various functionalities of nanocarriers for delivering drugs

#### Targeted nanocarriers

As is well-known, nanocarriers can achieve passive targeting of tumor tissues during PDIT via exploiting EPR effect 14, 38. Furthermore, by functionally modifying the nanocarriers, active targeting of tumor sites, including cell membrane targeting, mitochondrial targeting and TME targeting, can be successfully achieved for more tumor entry of nanomedicines. In this section, we will focus on nanocarriers with active targeting functions.

##### Cell membrane targeting

Some specific markers on the surface of various cancer cells, such as epithelial cell adhesion molecule (EpCAM) and type I transmembrane glycoprotein CD44, can be used for cell membrane targeting in tumor PDIT. EpCAM is highly expressed on the cell membrane of many types of cancer including hepatocellular carcinoma, colon cancer, lung cancer and gastric cancer 94, which mainly plays a crucial role in the signal transduction, proliferation and differentiation of tumor cells, and has been utilized for the development of PDT based on targeted micelles 95. Han et al. designed an EpCAM antibody-modified micelle loaded with mitoxantrone (MX) to treat hepatocellular carcinoma. Mice treated with these micelles showed significant fluorescence intensity, indicating that the EpCAM-conjugated micelles owned a good active targeting ability 57. As another tumor surface antigen, CD44 protein is highly activated during tumor invasion and metastasis 96, 97. Zhang et al. designed targeted BP NPs modified by PEGylated HA (HA-BP) for tumor PDIT 14, which could selectively accumulate at the tumor site through active targeting mediated by the HA receptor CD44. *In vitro* and *in vivo* experiments showed that HA-BP NPs had good biocompatibility, stability and therapeutic effect. Besides, the αvβ3 subtype in the integrin family, related to tumor proliferation, invasion and metastasis, is also highly expressed on the surface of various cancer cells 98. Hu et al. reported a αvβ3 receptor-targeted NP (MA-pepA-Ce6 NP) for PDIT 58. Of which, a small molecule PD-L1 inhibitor MET was covalently conjugated to Ce6 via a peptide linker (GPLGVRGDK, pepA). MA-pepA-Ce6 NP could quickly release MET and VRGDK-Ce6 after enzymatic response in TME. Subsequently, VRGDK-Ce6 could bind to the integrin αvβ3 receptor on tumor cell membrane to achieve tumor cell targeting, thus enhancing its tumor penetration and tumor cell internalization.

##### Mitochondrial targeting

It has been reported that, PSs can be delivered to mitochondria that may be more sensitive to ROS by using nanocarriers, thus resulting in better PDIT efficacy 99. The reason for this is that damaged mitochondria can increase the production of ROS and further show a greater tendency to trigger apoptosis 100. Accordingly, mitochondria have been considered as a preferred subcellular target in PDIT 101. As a representative mitochondrial-inclined lipophilic cation, alkyl TPP has been widely applied to modify some molecules such as PSs for improving the selectivity of mitochondrial absorption, and reducing the toxic effect on normal tissues. Wu et al. developed a mitochondrial-targeted graphene NP encapsulated with IR820 and CpG for tumor PDIT 61. In this study, TPP-modified graphene nanocarriers could specifically deliver IR820 into mitochondria and realize the photodynamic effect of mitochondrial localization. In another research, Peng et al. also prepared a TPP-based mitochondria-targeting nanocarrier for PDIT, where the positively charged polymeric micelle containing TPP groups was regarded as the core to load Ce6, and the charge transformational layer obtained from anionic 2,3-dimethylmaleic anhydride modified Biotin-PEG_4000_-NH_2_ via electrostatic interaction was regarded as the shell 74. Notably, the nanocarrier could expose TPP in tumor extracellular microenvironment, and the exposed TPP groups had the capacity of targeting the mitochondria of tumor cells, which facilitated Ce6 to generate ROS within mitochondria, thereby presenting an excellent antitumor effect in PDIT. Similarly, Yang et al. also employed a mitochondrial-targeted dual-loading system that covalently bound Ce6 and TPP-modified PEI to the CRISPR-Cas9 system targeting the Ptpn2 gene 10. As a result, the mitochondrial-targeting of nanocarriers can be expectably obtained by TPP modification during PDIT.

##### TME targeting

TME, containing the stromal cells, immune cells, vascular system, lymphatic system and extracellular matrix at the tumor site, usually plays a vital role in the occurrence, development and treatment of various cancers 102. Hence, it will be beneficial to improve PDIT effect by various targeting molecules-based nanocarriers delivering therapeutic cargoes to the components in TME, such as cancer-associated fibroblasts (CAFs), tumor-associated macrophages (TAMs), and tumor vasculature.

As a crucial component of TME, CAFs exist in diverse cancers 103, which affect tumor growth and metastasis. In addition, fibroblast activation protein (FAP), highly expressed on the surface of CAFs cell membrane, can become a useful tumor-targeting antigen for PDIT. For instance, Zhen et al. used FAP-specific single-chain variable fragments-modified FRT ZnF16Pc nanocage to achieve PDIT-based antitumor effect by active targeting of CAFs in TME 104. Also, TAMs are the most abundant leucocyte subset in many cancers and also play a vital role in boosting cancer progression 105. Actually, the elimination of TAMs by nanocarriers targeting their highly expressed surface receptors (e.g., mannose receptors) has been considered as a promising antitumor approach 106. Gao et al. reported a mannose-decorated mesoporous calcium silicate nanocomposite carrying indocyanine green (ICG) as a PS for PDT/PTT, which could obviously target TAMs in TME by the affinity between mannose and its receptor, thus effectively promoting tumor cell apoptosis 107. In addition, due to the overexpression of neuropilin-1 (NRP-1) on angiogenic endothelial cells, it is usually regarded as a common target of tumor vasculature-targeted therapy 108. Youssef et al. designed a gold nanorod loaded with PPa and “KDKPPR” peptide moiety for tumor PDT, which could achieve specific TME targeting by “KDKPPR” targeting NRP-1 of tumor vasculature, thus inducing a significant antitumor efficacy 109.

#### Stimulus-responsive nanocarriers

Generally, antitumor cargoes are first delivered to a specific tumor site by functional nanocarriers, and then quickly released in response to environmental stimuli. Notably, the stimulus-responsive drug delivery system can spatiotemporally control on-demand release of drugs, improve therapeutic efficacy and decline system toxicity 110. In this section, we will mainly emphasize the drug release of stimulus-responsive nanocarriers under internal stimuli (pH, redox potential gradient, enzymes) or external stimuli (photo response).

Among most stimuli, pH sensitivity is usually applied to trigger drug release 111. The acid-sensitive nanocarriers can trigger pH response in the TME (pH 6.8~7.2) or lysosome (pH 4.5~5.5) to release related drugs in PDIT 112, 113. In fact, pH-sensitive nanocarriers like liposomes have been gradually developed since the 1980s, which can be protonated in lysosomes to achieve pH response 114, 115. Yu et al. used pH-responsive nanoplatforms to deliver siRNA. In the TME with pH 6.8~7.2, acid-responsive micelles could be stimulated by pH to decompose and release siRNA for PD-L1 gene KD of tumor cells, thus enhancing the antitumor immune effect of PDIT 39. In addition, Yang et al. constructed a self-assembled smart nanovesicle composed of pH-responsive block copolymer (PEG-b-cationic polypeptide), which could realize the pH response under the lysosomal environmental stimulation of pH 4.5~5.5, to release the PS HPPH and IDO inhibitor IND, thus heightening the effect of PDIT 116. Indeed, this pH-sensitive smart nanovesicle offered great diversity and potential for the construction of nanomaterials for PDIT.

In addition to pH response, the intracellular redox potential gradient can also be served as a stimulus response to control drug release from nanocarriers. As a common reductant that acts on disulfide bonds, GSH is usually used in nano-drug delivery systems, whose concentration in the cytoplasm presents 0.5~10 mM 117. Once the disulfide bond is destructed by GSH, the delivered drugs can be instantly released to exert their effects. Xu et al. developed bMSN embedding TLR9 agonist CpG and Ce6 for tumor PDIT, which achieved a superior antitumor potency in PDIT by releasing immunostimulant CpG and antigenic peptides after breaking disulfide bonds 42. Moreover, this strategy was also used in multiple tumor-bearing mice models, demonstrating their potential to treat advanced cancers. Yu et al. used a GSH-responsive nanosystem-based PDIT strategy for the treatment of breast cancer 118. Specifically, the PPa and IDO-1 inhibitor NLG919 were linked with compounds containing disulfide bonds, and further loaded into OXA prodrugs to form the light-inducible nanocargo (LINC). After the LINC was intravenously injected and delivered into 4T1 tumor cells, disulfide bonds were broken due to redox response, further facilitating the release of PPa and NLG919 from LINC.

Enzymes, such as phospholipase and nitroreductase, can not only be applied as catalysts to participate in the organism's life activities, but also as responsive stimulants to achieve the controlled release of drugs from nanocarriers 119, 120. In a related research, a MMP-2-responsive multifunctional liposomal nanocarrier was introduced, where the MMP-2-sensitive linker could be disconnected by the catalysis of MMP-2 to release the protective long PEG segments 121. In addition, the functional application of enzyme response has been widely concerned in the studies of nanocarrier-based PDIT. For instance, Hu et al. prepared an enzymatically cleavable self-delivery NP (MA-pepA-Ce6 NP). Noticeably, pepA could be specifically cleaved by MMP-2 in TME, and then the photodynamic reagent Ce6 and PD-L1 inhibitor MET could be effectively released to eliminate breast cancer 58. Furthermore, Song et al. synthesized a chimeric peptide NP, which integrated PpIX with the checkpoint blocker IMT via a caspase-responsive peptide sequence Asp-Glu-Val-Asp. Once exposure to caspase stimulation, IMT could be easily released from decomposed nanocarriers to enhance tumor PDIT by activating antitumor immunity, ultimately effectively destroying primary and metastatic lung tumors 122.

Other external physical stimuli such as photo response have also been extensively explored for various nanodrug delivery systems. Usually, the light source can divide into ultraviolet light, visible light or NIR. The photosensitive nanocarriers loaded with therapeutic drugs can be cleaved to release drugs under the stimulation of the above light in PDIT. Sun et al. developed a ROS-sensitive polymeric nanocarrier to achieve light-controlled drug release, which enhanced the antitumor immune response of PDIT under NIR 123. Furthermore, Hu et al. explored a lipid-polymer hybrid nanocarrier with a ROS-responsive core for on-demand release of DOX under NIR, thus augmenting the efficacy of nanocarrier-based PDIT 124.

## Various strategies for enhancing PDIT effectiveness

Different types of nanocarriers (organic, inorganic, metallic, organic and inorganic composite nanocarriers) and their functionalities (targeting and stimulus response) have been summarized above in detail. Overall, the employment of nanocarriers can effectively ensure therapeutic efficacy and reduce off-target adverse reactions in the process of drug delivery. However, in recent years, several factors limiting the effectiveness of antitumor immunity in PDIT have attracted significant interest. As we known, O_2_-dependent PDT mainly produces inhibitory effects on tumors via cytotoxic ROS. Namely, when the ROS accumulates in large quantities within mitochondria, apoptosis-inducing factors can be further released to cause tumor cell death. Actually, higher O_2_ content can help the photodynamic nanosystem produce stronger antitumor inhibition effect 125. Whereas, both natural hypoxic state and PDT-mediated O_2_ depletion can results in low O_2_ concentrations of tumor area, subsequently contributing to an attenuated PDIT efficacy 126. Meanwhile, most conventional PDT strategies inducing tumor ICD cannot normally pose an apparent threat to tumor cells. In addition, although tumor cells can be recognized and killed by intrinsic T cells in the body's immune system, it has been found that tumor cells evolve the ability of immune escape, which can make T cells more difficult to effectively recognize the tumor cells, further resulting in a poor immune response. So, this negative regulation signaling has been often referred to as the immunosuppressive checkpoints 28, including PD-1/PD-L1, CTLA4 and IDO. Accordingly, it is vital to combine different checkpoint-blockade mechanisms to remove the constraints of these negative factors above, thereby reinforcing antitumor immune effect of PDIT.

Next, the underlying mechanisms of different immune-related drugs for enhanced tumor PDIT are detailedly illustrated. There are currently four main therapeutic modalities to enhance antitumor effectiveness of PDIT **(Table 2)**. First, to increase the O_2_ concentration in TME, delivering various O_2_ catalysts and oxygen carriers through the functional nanoplatforms can alleviate tumor hypoxia and increase the production of PDT-triggered ROS. Second, to boost tumor ICD effect, it is necessary to raise tumor ICD level and heighten the immunogenicity of tumor cells via combination strategy like PDT plus chemotherapy or PTT. After the significant activation of tumor ICD, the immune signal DAMPs secreted by dying tumor cells can further provoke the release of “eat me” signal of tumor 127, which can trigger the activation of the antitumor immune system for enhancing the antitumor immunity of PDIT. Third, to sufficiently stimulate immune response in PDIT, immunoregulatory adjuvants such as TLR agonists (R837 and CpG) can be integrated into nanocarriers, to promote the maturation of DCs and enhance immune responses that eradicate tumor cells. Last, to block immunosuppressive effect of checkpoints, PDT can be combined with ICB-based immunotherapy, in which ICIs, including anti-PD-1/PD-L1 antibodies, siRNA, anti-CTLA4 antibodies and IDO inhibitors, are usually embedded in NPs to effectively avoid the immune escape of tumor cells, ultimately inducing the death of tumor cells by immune-activated CTLs.

### Oxygen-increasing PDIT

The PSs can become excited states under light, which will generate free radicals or transfer energy to the surrounding O_2_ for producing ROS through a series of reactions, thus destroying tumor cells. Nevertheless, except for greatly restricting the antitumor efficiency of PDT, tumor hypoxia may even cause cancer recurrence 145. Especially, relevant studies in colorectal cancer cases showed that, there was a higher probability of tumor recurrence in cancer patients with hypoxia-induced fat mass and obesity-associated protein degradation 146. In recent years, much progress has been made in alleviating hypoxia in TME 147-149. It has been reported that, high concentrations of H_2_O_2_ in TME present a new opportunity for reversing the hypoxia-related drug resistance in tumor therapy 150, 151. Hence, some catalysts such as MnO_2_, cerium oxide (CeO_2_) and CAT, that own the capacity to catalyze the decomposition of endogenous H_2_O_2_ to produce O_2_, are usually introduced into nanosystems and widely applied for enhanced PDIT. In addition, directly delivering oxygen to the tumor site by utilizing PFCs or Hb can also effectively relieve hypoxia in TME, thus boosting the efficacy of PDIT.

**Figure 1:**
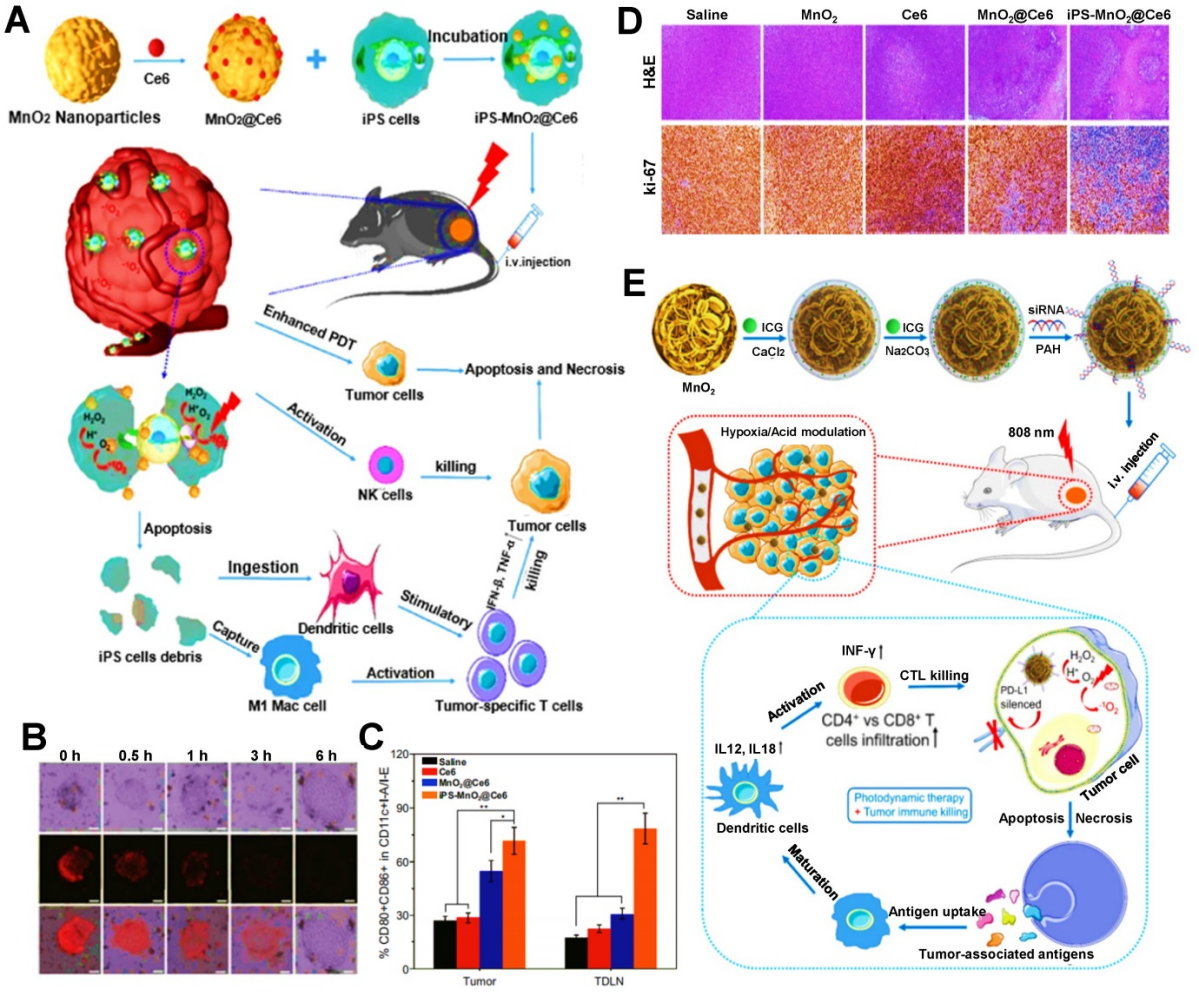
** (A)** Schematic representation of iPS-MnO_2_@Ce6-mediated antitumor photodynamic immunotherapy. **(B)** Confocal laser microscopy images of oxygen-sensing probe in iPS cells indicating the intracellular oxygen level incubated with nanoprobes for the certain time. **(C)** Mature DCs in tumor tissues and TDLN of staining with CD80 and CD86 for flow cytometry assay. **(D)** Images of H&E and Ki67 stained tumor slides from the mice after various treatments. Adapted with permission from 49, copyright 2020 Springer Nature Switzerland AG. **(E)** Schematic illustration of the mechanism of photodynamic tumor immunotherapy with MnO_2_ nanoparticles. Adapted with permission from 128, copyright 2019 Ivyspring International Publisher. Abbreviations: iPSs: induced pluripotent stem cells; ICG: indocyanine green; H&E: hematoxylin and eosin; CTLs: cytotoxic T lymphocytes; TDLN: tumor draining lymph node.

Previous studies have shown that, MnO_2_ exhibits a high degree of specificity and reactivity to H_2_O_2_, which can consume hydrogen ions to produce O_2_ and manganese ions 150, 152. Liu et al. reported a MnO_2_@Ce6 NP encapsulated in induced pluripotent stem cells (iPSs) for enhanced PDIT efficacy **(Figure 1A)** 49. The increasing O_2_ content in iPSs was found after measuring intracellular O_2_ levels with the oxygen-sensing probe **(Figure 1B)**, which also confirmed the catalytic effect of MnO_2_ on endogenous H_2_O_2_. Under laser irradiation, the photodynamic conversion effect of Ce6 induced a large amount of O_2_ to be converted into ROS, thus enhancing the suppressive effect of ROS on tumor cells due to the increase in the amount of ROS. In addition, after injecting different nanoformulations into each group of tumor-bearing mice, it was found that the proportion of DCs maturation in mice treated with iPS-MnO_2_@Ce6 was higher than that in other treatment groups **(Figure 1C)**. To certify the antitumor potency, after the tumor sections of each group were stained, the results showed that the tumor cell density in the iPS-MnO_2_@Ce6 group was the lowest** (Figure 1D)**. From these data, it could be seen that the generation of sufficient O_2_ not only remarkably eliminated tumors by promoting high production of ROS, but also exhibited the enhanced effect of anti-tumor immunity in PDIT. Besides, Pan et al. designed a CaCO_3_/MnO_2_ nanoplatform loaded with ICG and siRNA to enhance tumor PDIT** (Figure 1E)** 128. More specifically, the catalysis of H_2_O_2_ triggered by MnO_2_ augmented the O_2_ concentrations in TME, which further increased the contents of ICG-induced ROS under light, consequently raising the potency of antitumor immunity in PDIT. Besides, as common catalysts, CeO_2_ and titania (TiO_2_) are also widely used in PDIT. Zuo et al. constructed a mesoporous SiO_2_ NP co-loaded with CeO_2_, PS IR780 and MET, which generated enough O_2_ and Ce^2+^ after the etching of CeO_2_ in TME to boost IR780-mediated PDT, thus inducing a powerful antitumor PDIT effect 129. Additionally, for the catalyst TiO_2_, Zheng et al. prepared the Au@TiO_2_ core-shell NP carrying DOX, where TiO_2_ as a new-type PS was able to catalyze endogenous H_2_O_2_ to produce more O_2_, thus effectively inhibiting tumor cells by enhanced PDT 130.

**Figure 2:**
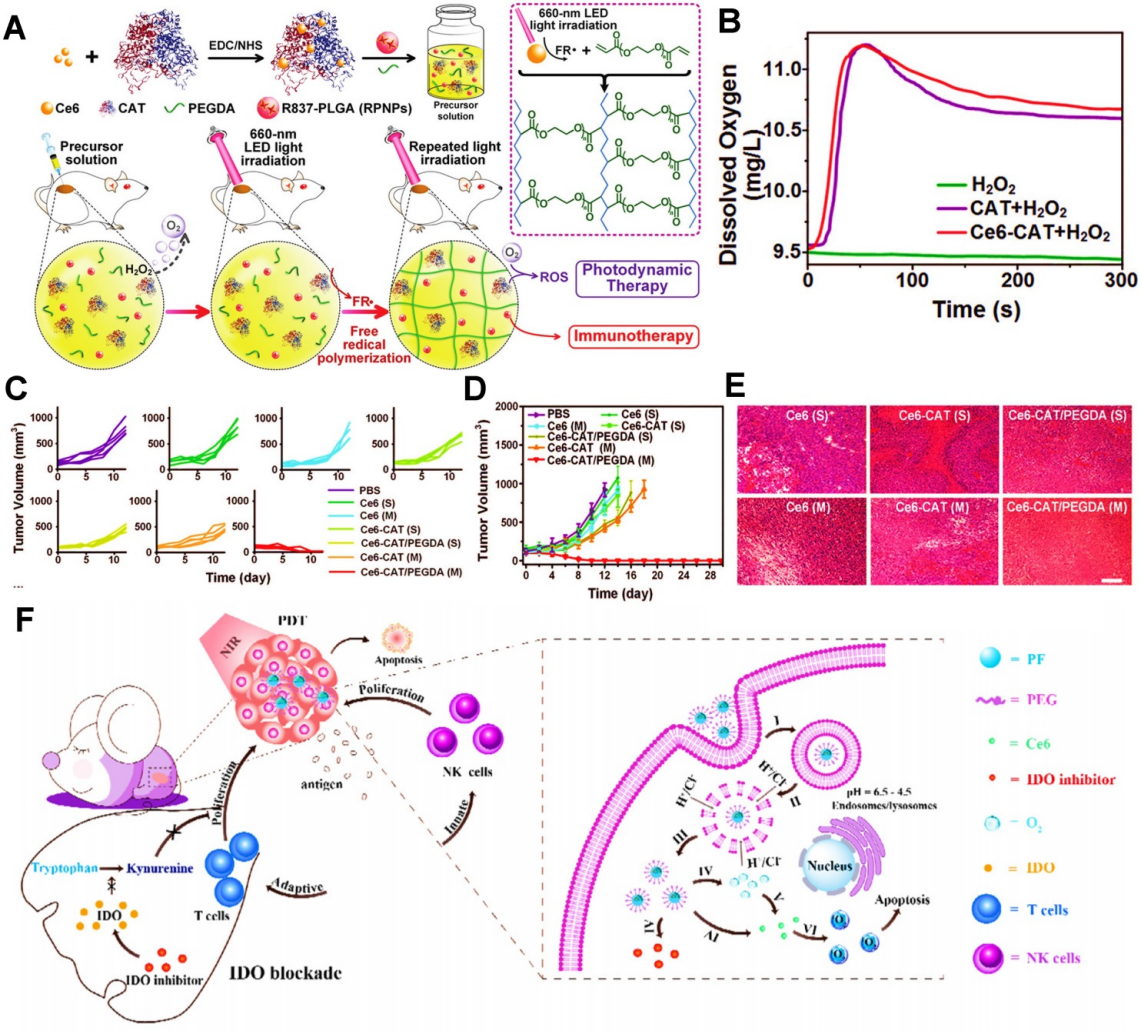
** (A)** The scheme showing the formation of Ce6-CAT/PEGDA hydrogel for applying in enhanced PDIT. **(B)** The dissolved oxygen generation by various formulations. **(C)** Individual and **(D)** average tumor growth curves of 4T1 tumor-bearing mice from various formulations. **(E)** Images of H&E stained tumor slices from diverse groups of 4T1 tumor-bearing mice. Adapted with permission from 32, copyright 2019 WILEY-VCH Verlag GmbH & Co. KGaA, Weinheim. **(F)** Schematic illustration of the mechanism of enhanced PDIT via utilizing PFCs. Adapted with permission from 132, copyright 2019 Elsevier Ltd. Abbreviations: CAT: catalase; PEGDA: PEG double acrylate; PFCs: perfluorocarbons; IDO: indoleamine 2,3-dioxygenase.

Except for the applications of MnO_2_, CeO_2_ and TiO_2_, CAT is also commonly used as a catalyst to ameliorate tumor hypoxia in PDIT. For instance, Meng et al. developed a PEG double acrylate (PEGDA) hydrogel (Ce6-CAT/PEGDA) combined with Ce6, immune adjuvant R837 and CAT for tumor PDIT 32. After hydrogel was injected into mice and exposed to 660 nm light, ROS induced by Ce6 could induce PEGDA polymerization **(Figure 2A)**.

In detail, this light-triggered *in situ* gel released CAT to break down endogenous H_2_O_2_ in tumor cells under light irradiation, thus resulting in increased O_2_ concentrations in TME. Therefore, these findings showed that, compared with the H_2_O_2_ group, both the CAT group and Ce6-CAT group could trigger more generation of O_2_ in the solution of H_2_O_2_ **(Figure 2B)**. When given light in the environment with sufficient O_2_, more ROS production was induced by Ce6, thus improving the ability to destroy tumor cells. In subsequent *in vivo* studies, the tumor volume and cell density in the Ce6-CAT/PEGDA group presented the smallest after 7 days of treatment **(Figure 2C-E)**, fully reflecting the augmented antitumor immunotherapeutic efficacy resulted from oxygen-increasing PDIT. Similarly, Yang et al. also prepared a kind of mitochondrial targeted/pH-responsive SiO_2_ NP integrated with CAT and Ce6 131. As expected, the catalytic effect of CAT increased intracellular O_2_ content of tumor cells, and then a large number of ROS induced by Ce6 was produced under 660 nm NIR, thus effectively enhancing the immunotherapeutic efficacy of PDIT. Furthermore, Shi et al. also reported a CAT-based liposome with PS MBDP and DOX, which could reverse immunosuppressive TME by CAT catalyzing intratumoral H_2_O_2_, thus strengthening killing effect on breast cancer during PDIT 153.

In addition, PFCs, as the inert chemical with extremely high O_2_ solubility, are capable of effectively storing oxygen molecules 154, which can be applied in PDIT to increase O_2_ concentration. For instance, Xing et al. constructed a fluorinated polymeric nanoparticle loaded with Ce6 and NLG919 (an IDO inhibitor) for synergistic tumor PDIT, which could induce stronger PDT efficacy by fluorinated polymers directly carrying high concentrations of oxygen and suppress immune escape of tumors by blocking IDO, thereby enhancing suppressive ability against breast cancer cells of PDIT **(Figure 2F)** 132. Aside from PFCs, Hb can also have a vital role in improving anoxic environment due to its high oxygen carrying capacity. Luo et al. prepared a tumor-targeted oxygen-carrying hybrid protein nanocarrier composed of Hb and albumin, which encapsulated DOX and Ce6 and dissolved a large number of O_2_ by Hb so as to produce enhanced PDT via strengthening O_2_ self-supply and ROS generation, thus leading to the effective elimination of tumors 155. Similarly, Chen et al. also fabricated a bioinspired hybrid protein oxygen nanocarrier containing Hb for improved PDIT, which could achieve the co-delivery of enough O_2_ and Ce6 to induce more sufficient PDT, ultimately evoking intense antitumor immunity 36.

### ICD-boosting PDIT

**Figure 3:**
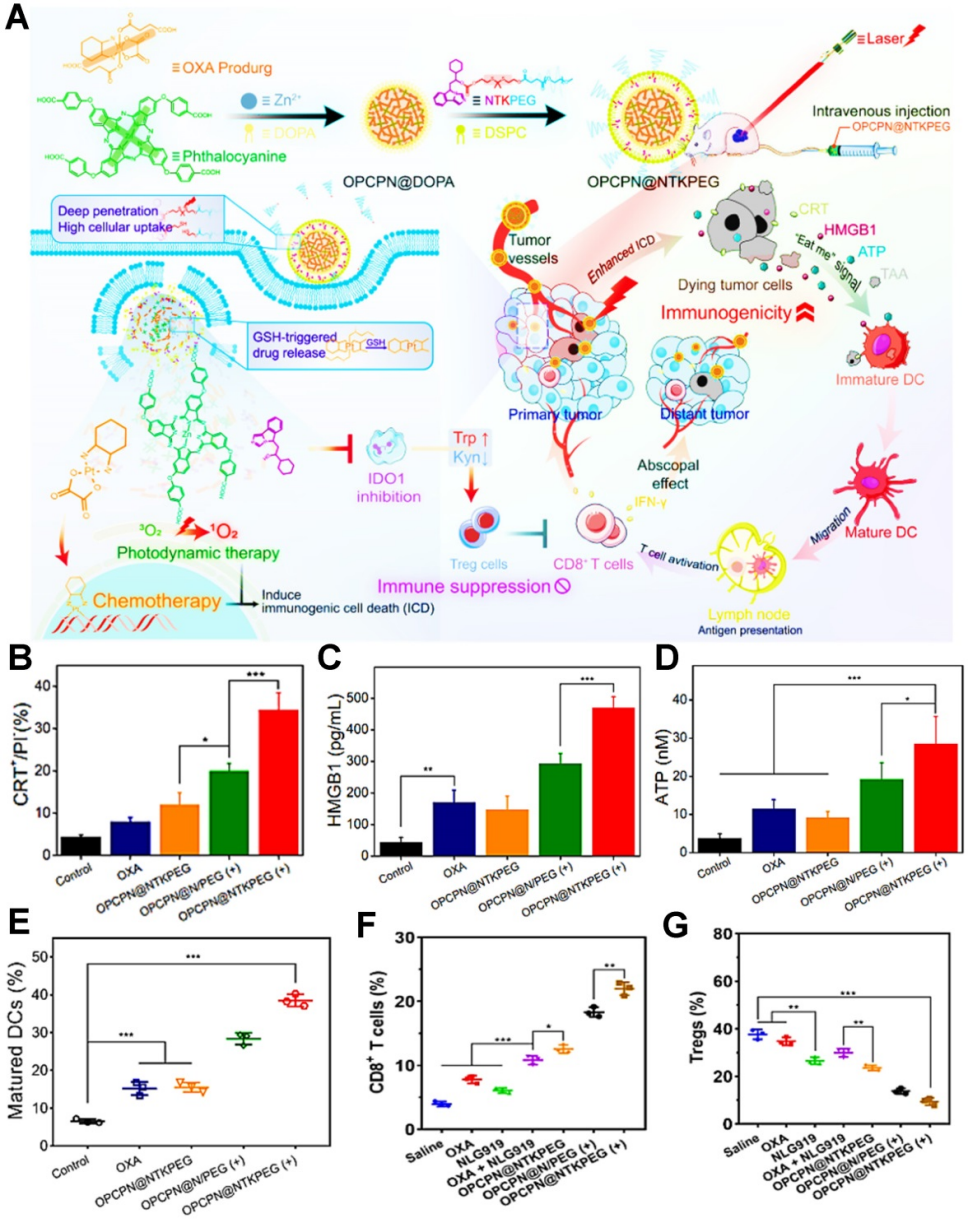
** (A)** Schematic illustration of PDT/chemotherapy combining with IDO inhibitor prodrugs to enhance antitumor efficacy. **(B)** Flow cytometric examination of CRT exposure. **(C)** Determination of HMGB1 release and **(D)** ATP secretion during antitumor PDIT. **(E)** Mature DCs ratio of tumor-bearing mice with different strategies. **(F)** Intratumoral infiltration ratio of CD8^+^ T cells and **(G)** Tregs in PDIT. Adapted with permission from 156, copyright 2021 American Chemical Society. Abbreviations: OXA: oxaliplatin; DOPA: dihydroxyphenylalanine; NTKPEG: NLG919-thioketal-PEG; Trp: tryptophan; Kyn: kynurenine; GSH: glutathione; OPCPN: oxaliplatin/phthalocyanine-based coordination polymer nanoparticle; CRT: calreticulin; HMGB1: high mobility group box 1; TAA: tumor-associated antigen.

During the tumor PDIT, ICD is characterized by releasing DAMPs-based immune signal from dying tumor cells. These DAMPs can interact with various receptors such as phagocytosis-related receptors, purinergic receptors and pattern-recognition receptors on the surface of innate immune cells to realize ICD-induced antitumor immune responses 5. However, tumor ICD activation is often limited by the low immunogenicity of tumors. Therefore, a series of ICD-boosting PDIT strategies such as the combination of PDT and chemotherapy have gradually attracted widespread attention. For instance, Huang et al. constructed a laser/GSH-responsive oxaliplatin (OXA)/phthalocyanine-based coordination polymer NP (OPCPN), which could achieve phthalocyanine-triggered PDT under laser irradiation and promote the ICD effect by OXA prodrugs exposing CRT, releasing HMGB1 and secreting ATP, further enhancing the antitumor immunity by PDT/chemotherapy combining with IDO inhibitor prodrug (NTKPEG) **(Figure 3A)** 156. To confirm PDT/chemotherapy could cause more powerful ICD, the research results suggested that, compared with the OXA and OPCPN@NTKPEG groups, OPCPN@NTKPEG (+) group-induced CRT exposure nearly increased 4.3-fold and 2.9-fold, respectively. Moreover, both HMGB1 release and ATP secretion from the tumor cells treated with OPCPN@NTKPEG (+) exhibited over 2 times as many as those from the groups of OXA and OPCPN@NTKPEG **(Figure 3B-D)**. In addition, the release of DAMPs obviously caused DCs maturation, subsequent tumor infiltration of CD8^+^ T cells and decline of regulatory T cells (Tregs), which resulted in the highest content of mature DCs, CD8^+^ T cells and minimum expression of Tregs in the OPCPN@NTKPEG (+) group compared to other groups **(Figure 3E-G)**, and eventually realized the best antitumor immunotherapeutic effect in PDIT. Likewise, Jin et al. designed a mixed nanocarrier based on UCNP as the core and SPTP micelle as the shell for enhanced PDIT, in which DOX and the PS rose bengal (RB) were integrated into this composite nanocarrier for boosting ICD effect by upregulating the expression of CRT and HMGB1 within tumors, thus dramatically augmenting antitumor immune response of PDIT 133. Yang et al. also synthesized a photo-responsive MSN co-loaded with chemo-drug DOX and PS methylene blue, which could amplify ICD effect by the synergistic PDT/chemotherapy under red light irradiation, subsequently evoking more powerful antitumor PDIT while combining with a PD-1 checkpoint blockade 157. Moreover, melittin (MLT) served as a common chemotherapeutic agent can be also employed for enhanced PDIT efficacy. For example, Liu et al. designed a serum albumin-coated boehmite encapsulated with Ce6 and a honey bee venom-based MLT peptide for improving PDT-mediated ICD levels 134. As a non-selective cytolytic peptide, MLT disrupted tumor cell membranes by forming transmembrane pores, which led to the initial damage of tumor cells. Furthermore, the formation of transmembrane pores by MLT also facilitated the accumulation of drugs in tumor cells and induced the expression and release of more DAMPs, thus markedly reinforcing the immune responses of PDIT.

**Figure 4:**
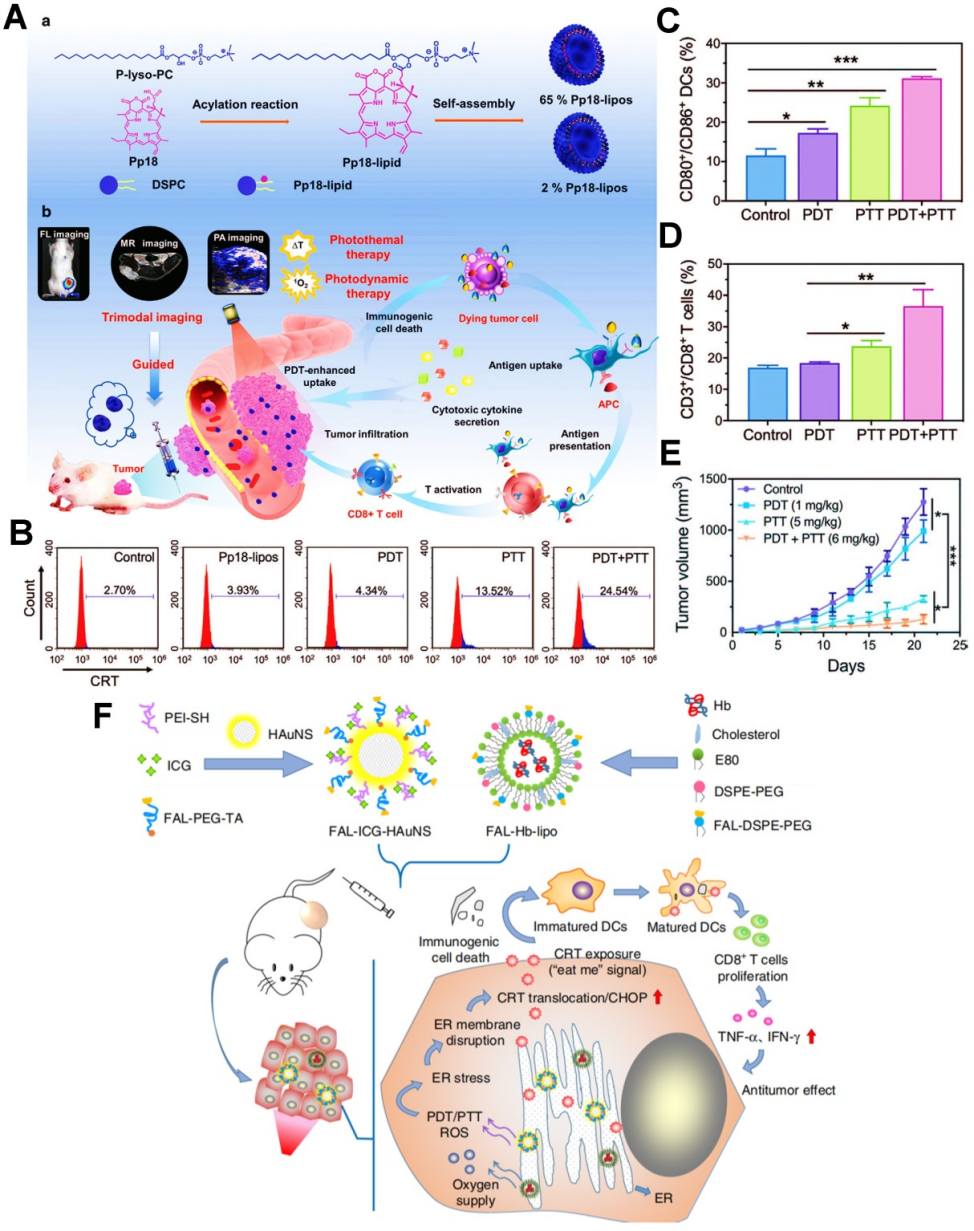
** (A)** Schematic illustration of the preparation of Pp18-lipos and application in enhanced PDIT. **(B)** Flow cytometry analysis of the level of CRT exposure after different phototherapies. **(C)** Frequency of tumor-infiltrating mature DCs in tumor-bearing mice with different treatments. **(D)** Corresponding quantification of CD3^+^/CD8^+^ T cells of mice by various treatments. **(E)** Tumor growth curves by different phototherapies. Adapted with permission from 135, copyright 2020 Wiley-VCH GmbH. **(F)** Schematic illustration of the formulation of nanocarriers and the mechanism of enhanced ICD via ER-targeting PDT/PTT. Adapted with permission from 43, copyright 2019 Springer Nature Limited. Abbreviations: ER: endoplasmic reticulum; Pp18: purpurin 18; PEI: polyethylenimine; HAuNS: hollow gold nanospheres; Hb: hemoglobin; E80: egg phosphatidyl lipid-80; TA: thioctic acid.

Furthermore, as an excellent antitumor immunotherapeutic strategy, the combination of PDT and PTT (PDT/PTT) based on nanomedicines also presents an apparent performance in exerting a potent ICD effect. In this regard, Sun et al. designed a versatile liposome-like nanoporphyrin carrying purpurin 18 as a PS for the synergistic PDT/PTT on the 4T1 tumor-bearing mice **(Figure 4A)** 135. Upon exposure to the 705 nm laser, the PDT effect could be carried out for generating a large amount of ROS, and meanwhile the purpurin 18 owned an excellent capability of photothermal conversion, thereby further achieving the collaborative antitumor efficacy of PDT/PTT. To further demonstrate the ICD-boosting effect, the flow cytometric analysis of CRT revealed that the level of CRT exposure on 4T1 cell membrane during the PDT/PTT had a nearly six-fold increase compared with applying PDT alone **(Figure 4B)**. On account of enhanced tumor ICD, it took the remarkable rise in the activation ratios of mature DCs and effector T cells **(Figure 4C, D)**. Indeed, the *in vivo* data proved that tumor cells could be noticeably suppressed because of the stronger immune responses provoked by PDT/PTT **(Figure 4E)**. Likewise, Li et al. established an endoplasmic reticulum (ER)-targeted nanosystem incorporating ICG and pardaxin peptides for PDT/PTT-mediated tumor immunotherapy, which further heightened tumor ICD followed by accelerated maturation of DCs and effective release of pro-inflammatory cytokines, thus ultimately exerting an excellent PDIT effectiveness **(Figure 4F)** 43. In another study, Liu et al. used a nanoneedle loaded with the PS aluminum phthalocyanine tetrasulfonate for enhanced tumor PDIT, which effectively facilitated the level of ICD via PDT/PTT-based combination therapy, thus resulting in high-efficiency treatment of tumors 136. As an aside, as distinguished from combination therapy, Deng et al. designed a reduction-sensitive polymeric NP loading ER-targeted PS TCPP-T^ER^ for the enhancement of PDIT via amplifying ICD 137. TCPP-T^ER^ could selectively accumulate in the ER of tumor cells upon NIR irradiation and locally induce ROS production that triggered oxidative stress of ER, which resulted in increased expression of CRT and HMGB1, and then elicited a stronger immune response of tumor suppression. Additionally, enhanced ICD can also be achieved by tailoring photophysical properties of PSs. Zhao et al. prepared a discrete ICG-loaded nanoaggregate by sterically hindered aggregation degree editor for improved tumor PDIT, which could concurrently alleviate aggregation-caused-quenching (ACQ) and photobleaching, thus evoking a powerful antitumor immune response by amplifying the ICD level of tumor 138.

### Adjuvant-promoted PDIT

**Figure 5:**
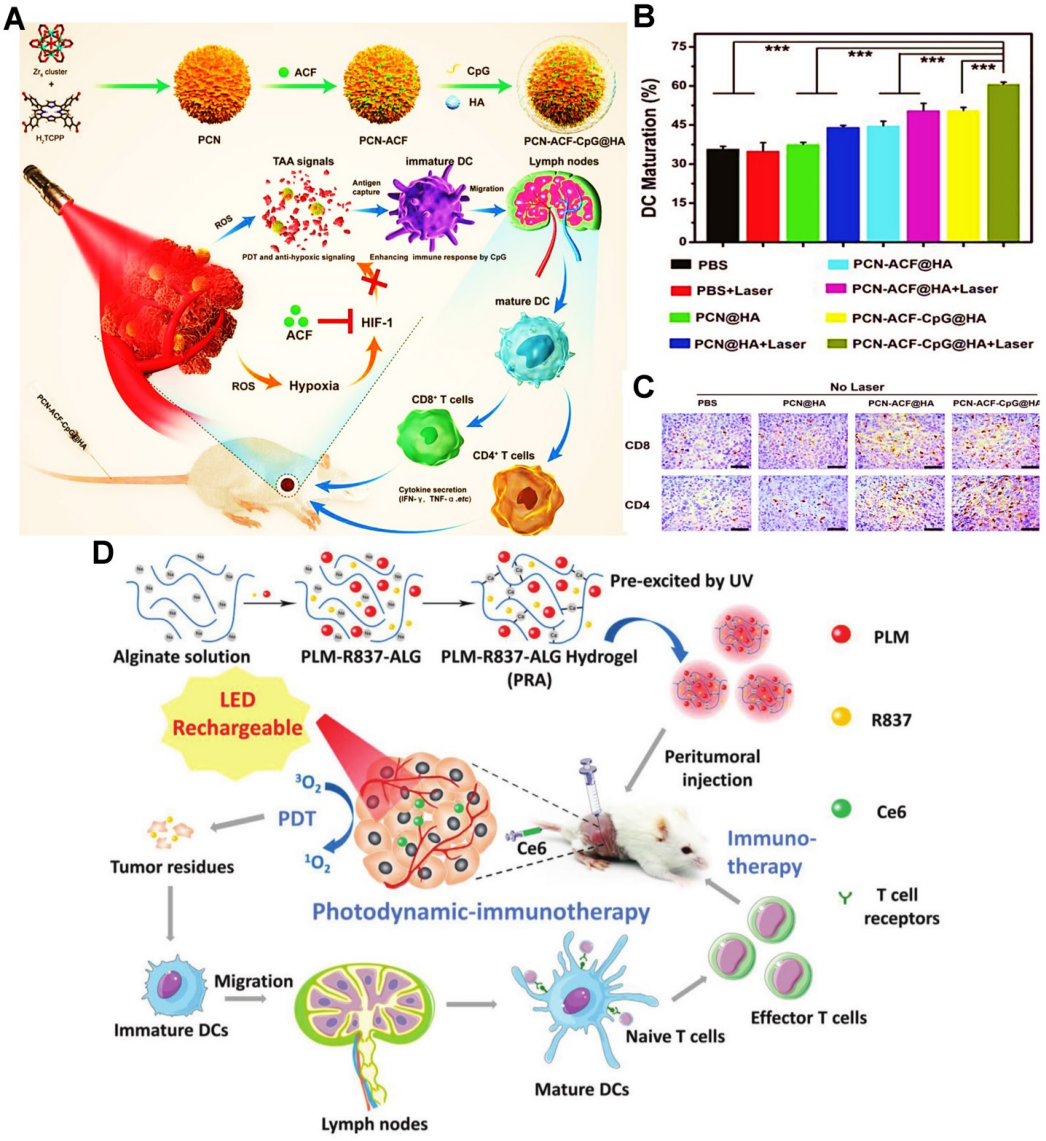
** (A)** Schematic illustration of the preparation of PCN-ACF-CpG@HA and principle for enhanced tumor PDIT. **(B)** Frequency of DCs maturation in tumor-bearing mice receiving different treatments by flow cytometry. **(C)** The CD4 and CD8 immunohistochemical images of tumors by using various formulations. Adapted with permission from 33, copyright 2019 WILEY-VCH Verlag GmbH & Co. KGaA, Weinheim. **(D)** Schematic illustration of the fabrication procedure and application of PLM-R837-ALG hydrogel during PDIT. Adapted with permission from 45, copyright 2021 Wiley-VCH GmbH. Abbreviations: ACF: acriflavine; H_2_TCPP: tetrakis (4-carboxyphenyl) porphyrin; TAAs: tumor associated antigens; TLR: toll-like receptor; HIF-1: hypoxia inducible factor-1; HA: hyaluronic acid; ALG: alginate; PLM: persistent luminescence material.

To improve the immunogenicity of antigens, the adjuvants can be supplemented to sub-unit or recombinant vaccines for boosting antitumor immunity 158. Inspired by this principle, some adjuvants like CpG or R837 can usually be encapsulated into nanocarriers for strengthening the antigen presentation of APCs during PDIT. For instance, Cai et al. reported a MOFs-based NP loading PS H_2_TCPP, adjuvant CpG and hypoxia-induced factor-1 inhibitor ACF for enhancing the effectiveness of tumor PDIT **(Figure 5A)** 33. During the PDIT, tumor cells were effectively eradicated by ROS under 670 nm laser irradiation, followed by the abundant release of tumor associated antigens (TAAs) and DAMPs. In terms of the level of DCs maturation within tumors, compared to other groups without laser irradiation or the treatment group of CpG, it was found that the PCN-ACF-CpG@HA group with laser irradiation exhibited the highest percentage (61.21%) of DCs maturation **(Figure 5B)**. The probable reason was that CpG released from MOF NP could activate TLR9 on the endosomal membrane of DCs, and further released a large number of cytokines to induce DCs maturation. Moreover, the ability of antigen presentation was also improved by the increased mature DCs, thus evoking a mass of activated T cells to eliminate tumor cells. As expected, the more tumor infiltration of CD4^+^ T cells and CD8^+^ T cells were distinctly observed in the PCN-ACF-CpG@HA group under laser irradiation **(Figure 5C)**. Similarly, Wen et al. designed thiol-activated bovine serum albumin NPs (TABNs) for tumor PDIT 140. Specifically, TABNs were first anchored onto the surface of tumor cells, and then the thiol-exposed BSA molecules were introduced to link TABNs to an albumin-based net that spatially caged tumor cells. In addition, Ce6 and CpG could be individually attached onto the tumor cell surface through hydrophobic and electrostatic interactions. The immunoregulatory CpG was further applied to activate TLR9, thus maintaining immunostimulation by increased expression of heat shock protein 70 (HSP70) and continuous exposure of tumor antigens. The final results showed that the strong and persistent immunostimulation promoted the sufficient maturation of activated CTLs, leading to the enhancement of immunotherapeutic effect in PDIT. In another research, Shu et al. proposed an enhanced PDIT strategy, namely that a hydrogel loaded with Ce6 and adjuvant R837 was fabricated for improving adaptive immune responses against tumors **(Figure 5D)** 45. R837 regarded as a TLR agonist could specifically activate TLR7 on the lysosome membrane, thereby heightening the immunogenicity of TAAs. As a consequence, it was demonstrated that a big number of mature DCs could be activated to accelerate the antitumor immune response by that strategy above.

### ICB-combined PDIT

At present, ICB-based immunotherapy has become a first-line treatment option for most cancers 159, 160. It is worth noting that PDIT combined with ICB can produce a synergistic effect and enhance the response rate of ICIs 152, 161, thus producing the optimal antitumor potency of PDIT.

#### PD-1/PD-L1 blockade

**Figure 6:**
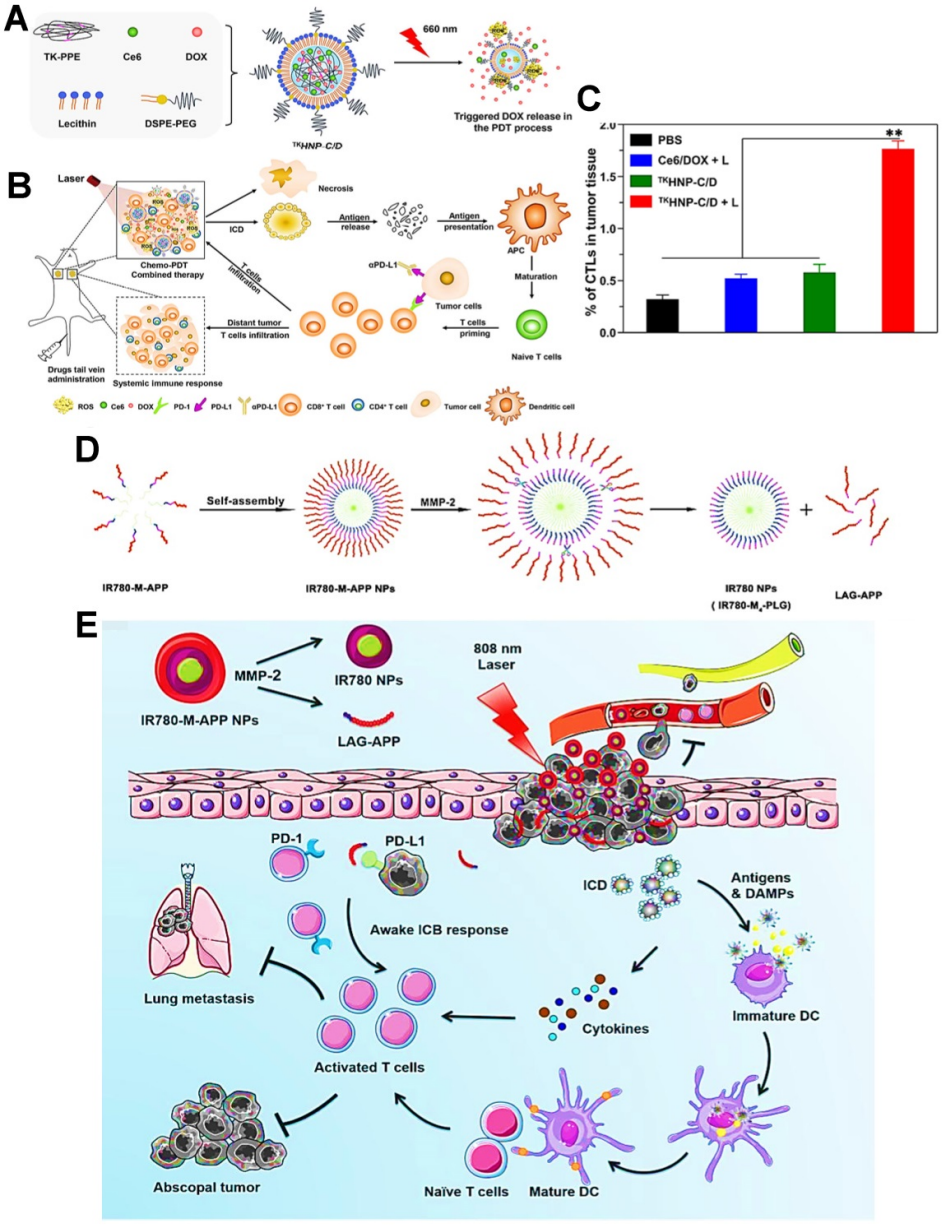
** (A)** Schematic representation of triggered DOX release from TKHNP-C/D under 660 nm laser. **(B)** The working principle of antitumor immune responses in PDT combined with PD-1/PD-L1 blockade therapy. **(C)** Flow cytometric analysis of the intratumoral infiltration of CTLs by various treatments. Adapted with permission from 124, copyright 2019 Elsevier Ltd. **(D)** The preparation and MMP-2 response of IR780-M-APP NPs. **(E)** Schematic illustration of antitumor immune responses based on combination of PDT and ICB immunotherapy under 808 nm laser. Adapted with permission from 142, copyright 2020 Elsevier B.V. Abbreviations: TK-PPE: thioketal phosphoester; MMP-2: matrix metalloproteinase-2; APP: anti-PD-L1 peptide.

As a common immune checkpoint, the PD-1 receptor on the surface of the T cell membrane can bind to the PD-L1 on the surface of the tumor cell membrane, which directly impedes the activity of T cells and prevent T cells from attacking tumor cells 162. To avoid this issue, a variety of therapeutic strategies have been well studied for this type of ICB, among which the blocking strategy based on PD-1/PD-L1 immune checkpoint holds great potential 142. Indeed, the anti-PD-1/PD-L1 antibody has been currently considered as an effective ICI for cancer immunotherapy. For anti-PD-1 antibody, Gao et al. used an integrin αvβ6-targeted phthalocyanine dye-labeled probe combined with anti-PD-1 antibody-based ICB for the synergistic tumor PDIT. During this combination strategy, phthalocyanine dye-labeled probe could evoke powerful antitumor efficacy of PDT by generating sufficient ROS under 690 nm laser, and anti-PD-1 antibody could achieve the specific blockade of checkpoint PD-1 for impeding immune escape of tumors, ultimately effectively suppressing breast cancer growth and lung metastasis 141. In terms of anti-PD-L1 antibody, Hu et al. reported lipid-polymer hybrid NPs loaded with Ce6 and DOX, which could finally achieve enhanced effectiveness of PDIT under light irradiation **(Figure 6A)** 124.

In this system, ROS-based photodynamic immunogenicity induced by Ce6 and tumor immunogenicity elicited by DOX could lead to a certain antitumor immunotherapeutic effect that was still blocked by PD-1/PD-L1 pathway. Accordingly, to strengthen specific T cells-based immune response during PDIT, the anti-PD-L1 antibody was further adopted and injected into tumor-bearing mice. As expected, the anti-PD-L1 antibody could effectively bind to PD-L1 on tumor cell membrane, and then competitively block the interaction between PD-L1 and PD-1 **(Figure 6B)**, thus facilitating the sufficient tumor infiltration of activated CTLs that owned the ability to eliminate tumors **(Figure 6C)**. In this research, PDIT combined with anti-PD-L1 antibodies exhibited a powerful systemic immune response, resulting in obvious elimination of both primary and distant tumors 44. Also, Wang et al. proposed an ICB-combined PDIT strategy for PD-L1 blockade by using anti-PD-L1 peptide instead of anti-PD-L1 antibody 142. In detail, a MMP-2-responsive polymeric NP co-loading the PS IR780 and anti-PD-L1 peptide was constructed for improved PDIT **(Figure 6D)**. Under 808 nm NIR irradiation, the photodynamic conversion of IR780 enabled PDT-induced tumor ICD by generating ROS. More importantly, the anti-PD-L1 peptide released from NP specifically blocked checkpoint PD-L1, leading to the improved effect of PDIT on destroying tumor cells and suppressing lung metastasis of tumors **(Figure 6E)**.

**Figure 7:**
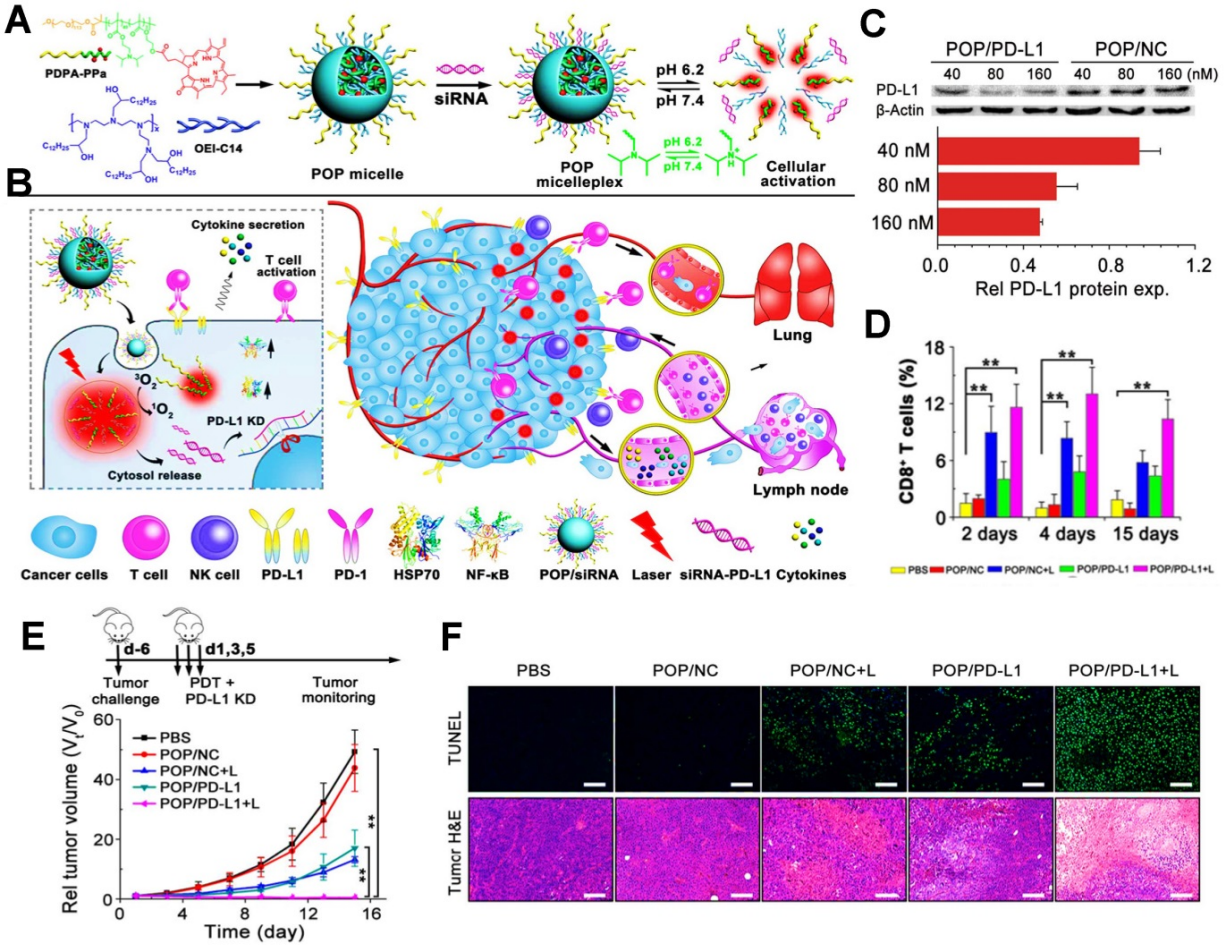
** (A)** Schematic illustration of preparation of POP micelleplexes loaded with PPa and siRNA and pH response in the acid surrounding. **(B)** Schematic drawing of POP/PD-L1 micelleplex-mediated tumor PDIT. **(C)** Western blot assay of PD-L1-KD in B16-F10 cells after receiving different POP micelleplexes loaded with 40, 80, 160 nM siRNA, respectively. **(D)** Proportion of tumor-infiltrating CD8^+^ T cells during PDIT. **(E)** Tumor growth inhibition curves by various strategies. **(F)** Images of TUNEL and H&E staining of the primary tumors. Adapted with permission from 39, copyright 2016 American Chemical Society. Abbreviations: siRNA: small interfering RNA; KD: knockdown; NF-κB: nuclear factor kappa B; PBS: phosphorus buffer saline; TUNEL: terminal deoxynucleotidyl transferase-mediated dUTP nick- end labeling.

In addition, PDIT strategy can also combine with siRNA to block the immune checkpoint PD-L1 for the elimination of tumor cells. For instance, Wang et al. used an acid-activatable micelleplex nanoplatform (POP micelles) carrying the PS PPa and PD-L1 checkpoint-blocked siRNA, which could further enable the enhanced effect of anti-tumor PDIT **(Figure 7A, B)** 39. In a further study, to prove the ICB potency by PD-L1 KD, Wang et al. prepared the POP micelles loaded with siRNA/PD-L1 (POP/PD-L1) and POP/NC for synergistic PDIT. Indeed, it was found that POP/PD-L1 could present stronger capacity of PD-L1 KD with increasing concentration of siRNA, thus dramatically inhibiting PD-L1 expression. Particularly, while the concentration of siRNA in the POP micelles remained 160 nM, more than 50% of PD-L1 expression presented downregulated **(Figure 7C)**. During tumor PDIT, siRNA-caused PD-L1 downregulation resulted in the inability of the tumor cell membrane surface to produce PD-L1 ligands, thus indirectly blocking PD-L1 followed by an increase in activated T cell** (Figure 7D)**. In *in vivo* assessment of antitumor potency, compared to other groups, POP/PD-L1 with laser irradiation group completely eliminated the B16-F10 tumors without body weight loss of mice **(Figure 7E)**. Moreover, TUNEL and H&E staining of the tumor sections showed that PDT combined with PD-L1 KD significantly induced apoptosis of the tumor cells **(Figure 7F)**, which reflected the stronger antitumor effect generated by the cooperation with ICB-based immunotherapy.

#### CTLA4 blockade

**Figure 8:**
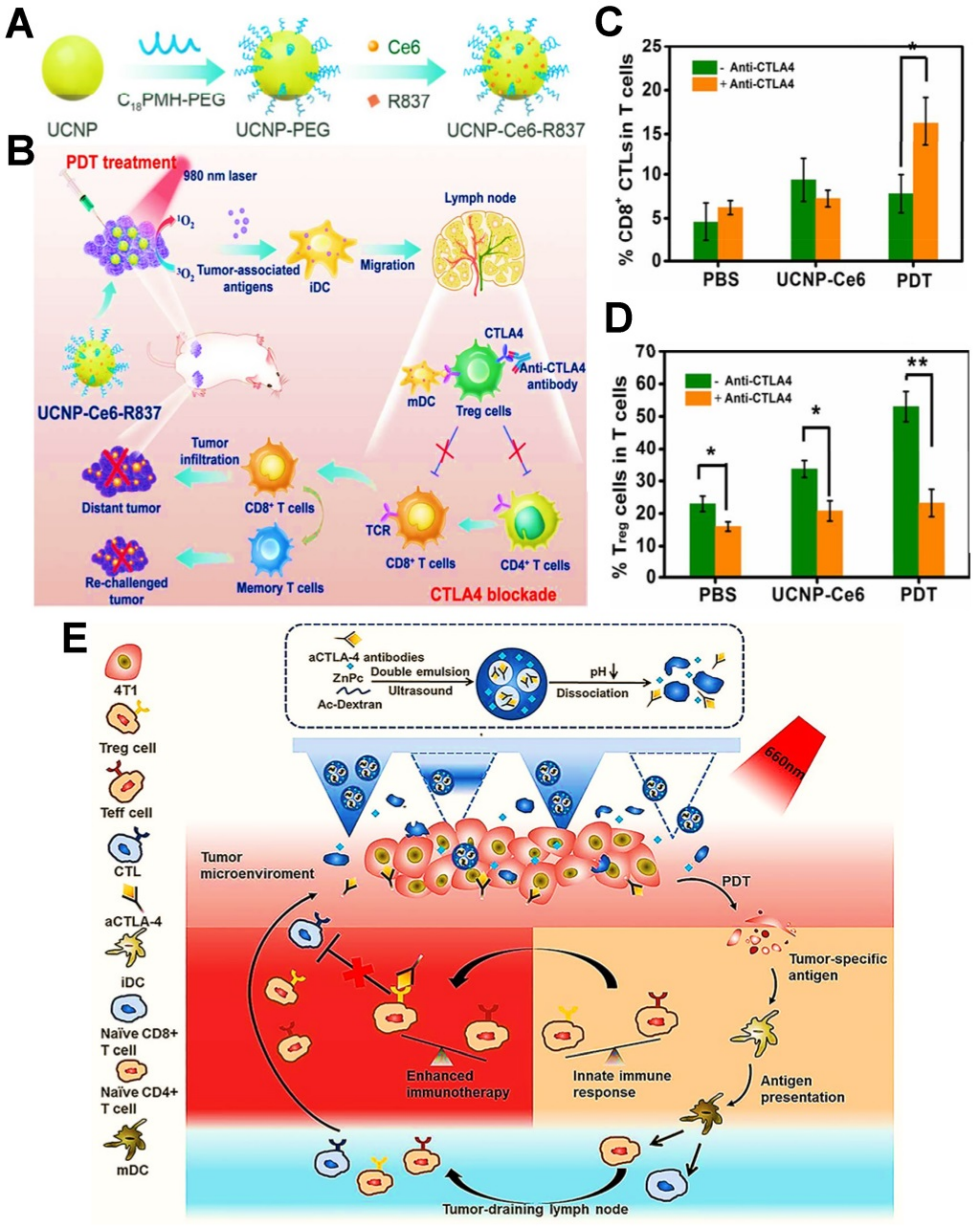
** (A)** Schematic illustration showing the fabrication procedure of UCNP-Ce6-R837. **(B)** Mechanism of enhanced cancer PDIT by combining NIR-mediated PDT with CTLA4 checkpoint blockade therapy. **(C&D)** Frequency of tumor-infiltrating CD8^+^ CTLs (C) and Tregs (D) in the distant tumor. Adapted with permission from 9, copyright 2017 American Chemical Society. **(E)** Scheme of the co-delivery system loaded with CTLA4 antibodies and mechanism of antitumor immune responses induced by PDT in combination with CTLA4 blockade. Adapted with permission from 143, copyright 2020 Elsevier B.V. Abbreviations: UCNP: upconversion nanoparticle; TCR: T cell receptor; mDC: mature dendritic cell; ZnPc: zinc phthalocyanine.

During the immune response of PDIT, in addition to the engagement of antigen-major histocompatibility complex (MHC) complexes with T cell receptors, extra costimulatory signals are also necessary for T cell activation 163, 164. Among them, B7 molecules expressed on APCs (e.g., DCs, B cells) and CD28 on T cells are usually identified as two important costimulatory molecules, which can generate costimulatory signals by mutual combination 165. Notably, as a second counter-receptor for the B7 family of costimulatory molecules, CTLA4 can negatively regulate the activation of T cells by displacing CD28 costimulation 166, thus suppressing antitumor immunity. Accordingly, the anti-CTLA4 antibody can be usually considered as a common ICB agent for improved PDIT, which can inhibit tumor immune escape by effectively blocking CTLA4 on the surface of T cells, further activating a large number of CD8^+^ T cells to eliminate tumors. For example, Xu et al. designed an UCNP that co-loaded the Ce6 and the adjuvant R837 for combined PDIT **(Figure 8A)** 9. While injected into mice and given light, the NPs triggered PDT for destroying tumor cells. Subsequently, after injection of anti-CTLA4 antibodies into mice, CTLA4 molecules on the surface of T cells could effectively bound to anti-CTLA4 antibodies, which prevented CTLA4 from competitively binding to mature DCs, thereby enabling the increased levels of CD8^+^ T cells and decreased levels of Tregs for boosting antitumor effect **(Figure 8B-D)**. Additionally, Chen et al. constructed a pH-responsive dextran NP co-loaded with the PS zinc phthalocyanine and anti-CTLA4 antibody to apply in 4T1 tumor-bearing mouse models, which could induce an abundant activation of T cells through blocking CTLA4, effectively eliminating breast tumor cells during PDIT **(Figure 8E)** 143.

#### IDO blockade

**Figure 9:**
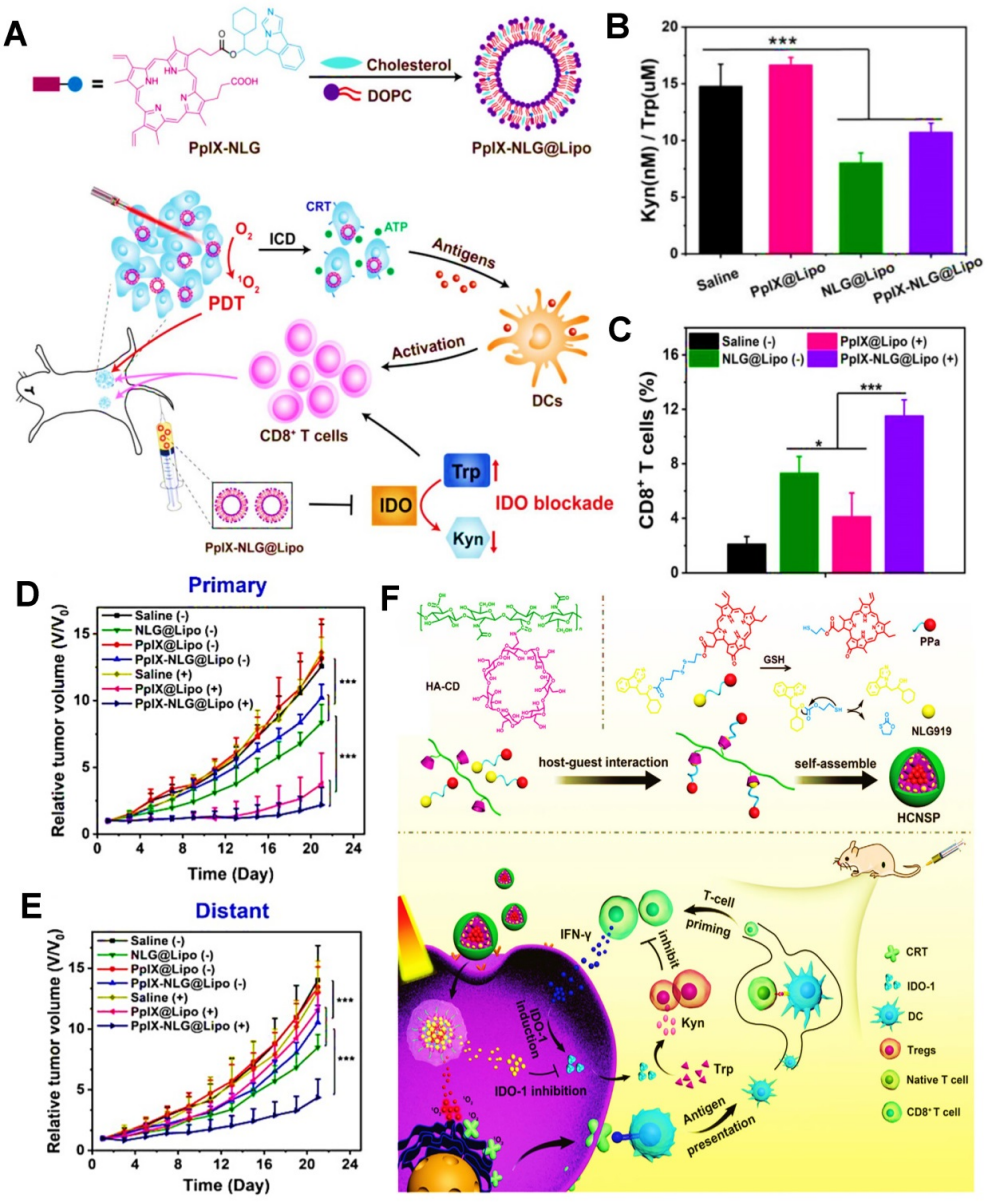
** (A)** Schematic illustration of preparation of PpIX-NLG@Lipo and mechanism of enhanced PDIT. **(B)** Kyn/Trp ratios in plasma of 4T1 tumor-bearing mice by different formulations. **(C)** Frequency of CD8^+^ T cells infiltration in the distant tumors detected by flow cytometric analysis. **(D)** Primary and **(E)** distant tumor growth curves of mice after receiving different treatments during PDIT. Adapted with permission from 47, copyright 2019 Ivyspring International Publisher. **(F)** Schematic illustration of fabrication of HCNSP nanocarrier via host-guest interaction and working principle of combination antitumor immunotherapy by simultaneous ICD induction and IDO-1 inhibition. Adapted with permission from 144, copyright 2020 WILEY‐VCH Verlag GmbH & Co. KGaA, Weinheim. Abbreviations: PpIX: protoporphyrin IX; Kyn: kynurenine; Trp: tryptophan; HA: hyaluronic acid; CD: cyclodextrin; CRT: calreticulin.

Unlike other immune checkpoints, IDO belongs to a special class of small molecule inhibitors 167, such as NLG919. Endogenous IDO is often defined as an immune-mediated enzyme that can catalyze the oxidative metabolism of tryptophan (Trp), thus accelerating the degradation of Trp into kynurenine (Kyn). Actually, the lack of Trp can usually impair the activity of CTLs, and conversely, the accumulated Kyn can heighten the activity of Tregs 168-170, thus causing a certain inhibitory effect on cancer immunotherapy. From this principle, IDO blockade becomes a feasible strategy for improved antitumor immunity in PDIT. Huang et al. constructed a liposome co-loaded with PpIX and NLG919, which could achieve the synergistic effect of PDT and IDO-based ICB, and thus apparently restraining tumor growth** (Figure 9A)** 47. In this research, PpIX released from liposomes was able to destroy tumor cells by the production of ROS under light, and meanwhile, immune checkpoint IDO could be successfully blocked by small molecular NLG919. Compared with the saline and PpIX@Lipo groups, the Kyn/Trp ratios were dramatically reduced and more CD8^+^ T cells were generated following the treatments of NLG@Lipo and PpIX-NLG@Lipo groups** (Figure 9B, C)**, implying that IDO was significantly inhibited by NLG919 and thus effectively stimulating T cell immunity. Notably, due to PDIT combined with IDO-blockade, it was discovered that the PpIX-NLG@Lipo with light irradiation group revealed the most CD8^+^ T cells and obvious inhibitory effects on both primary and distant tumors, eventually resulting in a prominent reduction in tumor volume **(Figure 9D, E)**. Likewise, Hu et al. also designed a GSH-responsive HA NP combined with Ce6 and NLG919 via host-guest interaction for enhanced PDIT, which expectably brought about a superior antitumor immune response by the combination of Ce6-based PDT and NLG919-induced IDO blockade **(Figure 9F)** 144.

## Conclusion and outlook

In recent years, the combination strategy of PDIT has attracted more and more attention for its promising application in the treatment of tumor recurrence and metastasis. With the rapid development of nanomedicine, a variety of multifunctional nanocarriers have been developed for antitumor PDIT. It should be noted that different types of nanocarriers have diverse structural characteristics and functions. In general, therapeutic payloads are loaded into different delivery nanoplatforms through physical hydrophilicity, hydrophobic interaction or electrostatic adsorption. During drug delivery, functional nanocarriers can be utilized to precisely target the tumor cells and responsively release the therapeutic cargoes to target site upon exposure to various physical or chemical stimuli, thus exerting the corresponding antitumor effect. Whereas, as a result of severe hypoxia, poor immunogenicity and immune escape in TME, the tumor-suppression effect of PDIT is extremely limited. Therefore, it is necessary to consider combining different treatment strategies, including increasing O_2_ concentration, boosting ICD effect, enhancing tumor immunogenicity, to activate mature DCs and inhibit the immune escape of tumor cells, thus strengthening the anti-tumor immune responses of PDIT.

However, there are still many challenges in anti-tumor PDIT. First, some certain nanomaterials own inherent toxicity, which may produce obvious inhibitory effects on other normal tissue cells in the body. Hence, we need to design more hypotoxic nanomaterials such as liposomes to ensure systematic safety during tumor PDIT. Second, due to the insufficient light penetration, the PSs cannot be effectively stimulated for more production of ROS, thus leading to a great impact on the efficacy of PDIT. At present, some high penetration light sources like X-rays have been utilized to enhance PDIT by augmenting photodynamic conversion efficiency 171. Even so, there is a still concern here that high penetration light-based PDIT should ensure the less damage to normal tissues. Furthermore, it may become a feasible strategy that PSs with high photoconversion capacity, such as IR780 or RB, can also be abundantly developed for improved tumor PDIT 172, 173. Third, to a certain extent, tumor hypoxia can also limit the efficacy of oxygen-driven PDT. Usually, we can solve this problem by nanoformulations loading oxygen catalysts (e.g., CAT and MnO_2_) or oxygen carriers (e.g., PFCs and Hb). The underlying mechanisms of these catalysts and oxygen carriers were concretely discussed in Section “Oxygen-increasing PDIT”. Fourth, immune escape of tumor cells can directly contribute to a poor antitumor immune response, which also become a tough challenge in tumor PDIT. To address it, diverse strategies, especially ICB-combined therapy, have been extensively developed for preventing checkpoints-based immunosuppression of tumor. In addition, the development of new-type immune checkpoints and relevant inhibitors may bring about a more powerful anti-tumor immune response, which also requires extensive laboratory exploration and research. Fifth, in basic or clinical studies, ICB-based antibodies such as anti-CTLA4 and anti-PD-1/PD-L1 are usually administered individually instead of the development of nano-preparation, one possible reason attributed to a certain decrease in antibody activity during NP delivery. Thus, it is urgent to develop different functional some small molecule reagents such as siRNAs or compounds to replace macromolecule ICB antibodies, which may hold great potential in nanotherapeutics-based PDIT for achieving the effective delivery of ICIs.

Although some advances have been achieved in basic research on tumor PDIT, it still faces some difficulties in its clinical transformation. First of all, the antitumor effect of PDIT exhibits as poorly as that of clinical traditional treatments. Additionally, it is difficult to accurately control the light conditions that trigger the photodynamic conversion of PSs in clinical practice. What's more, the anti-tumor effect of PDIT is mostly evaluated in ordinary mice, which makes us doubt whether PDIT can also have a strong therapeutic effect on clinical patients. So more researches should be assessed in humanized mice, non-human primates, pigs and so on. Altogether there are many limitations in preclinical studies of PDIT, one thing for sure is that PDIT can be considered as an effective and minimally invasive strategy for tumor therapy, which exhibits good clinical development prospects in colorectal cancer, breast cancer, lung cancer, skin cancer, etc. 174-177. Therefore, there is an urgent need for improvement and extensive studies in light conditions, animal models, material design, and drug combinations. It is deeply believed that PDIT can play a central role in the clinical cancer therapy in the future.

## Figures and Tables

**Table 1:** Various nanoformulations for delivering therapeutic reagents during PDIT

Category	Nanoformulation	Reagent	Drug-loading mechanism	Targeting ligand/receptor	Stimulus response	Target	Refs.
Organic nanocarrier	Liposome	Ce6/IPI-549	Hydrophobic forces	IPI-549/PI3Kγ	pH	Colon cancer	55
		Gemcitabine	Hydrophilic interaction	/	Photo	Biliary tract cancer	56
	Polymeric micelle	Mitoxantrone	Hydrophobic forces	Anti-EpCAM/EpCAM	Photo	Liver cancer	57
		PPa/siRNA	Covalent binding/Electrostatic absorption	siRNA/PD-L1	pH	Melanoma	39
	Polymeric nanoparticle	MET/Ce6	Hydrophobic forces	VRGDK/Integrin αvβ3	Enzyme	Breast cancer	58
		IR-780/IMT/GITR	Electrostatic absorption/Hydrophobic forces	GITR antibody/Treg cells	pH	Melanoma/Colon cancer	48
		JQ1/PPa/HA	Host-guest interaction	HA/CD44	Redox	Pancreatic cancer	46
		PpIX/PCPK	Hydrophobic forces	PCPK/PM	Enzyme	Breast cancer	59
Inorganic nanocarrier	GQDs	Ce6/HA	Covalent binding	HA/CD44	Photo	Lung cancer	60
	Graphene	IR820/CpG/TPP	Hydrophobic forces	TPP/Mitochondrion	Photo	Breast cancer	61
	BP	HA	/	HA/CD44	Photo	Leukemia	14
Metallic nanocarrier	Fe_3_O_4_ nanoparticle	Ce6	Covalent binding	cell membrane/tumor tissue	Redox/pH	Breast cancer	62
Organic and inorganic composite nanocarrier	NCP	OXA/Pyrolipid	Hydrophobic forces	/	/	Colorectal cancer	44
	UCNP	Ce6/R837	Hydrophobic forces	/	/	Colorectal cancer	9
	MOF	H_2_TCCP/CpG/HA	Coordination effect/Electrostatic absorption	HA/CD44	Enzyme	Liver cancer	33

**Table 2:** Various strategies for enhancing the antitumor efficacy of PDIT

Strategy	Nanoformulation	Reagent	Mechanism of enhanced PDIT	Target	Refs.
Oxygen-increasing PDIT	MnO_2_	ICG and siRNA	Generate more oxygen by the catalysis of MnO_2_ and silence; PD-L1 by siRNA	Lung cancer	128
	MSN	CeO_2_, IR780 and MET	Alleviate hypoxia in tumor by the etching of CeO_2_ in TME	Melanoma	129
	Au@TiO_2_	DOX	Produce enhanced PDT by TiO_2_ catalyzing H_2_O_2_ to release more oxygen	Cervical cancer	130
	SiO_2_	Ce6 and CAT	Overcome tumor hypoxia by CAT triggering decomposition of tumor endogenous H_2_O_2_	Breast cancer	131
	Polymeric nanoparticle	Ce6 and NLG919	Elicit stronger PDT efficacy by fluorinated polymers directly carrying O_2_	Breast cancer	132
	Hybrid protein oxygen nanocarrier	Ce6	Induce more sufficient PDT by Hb directly delivering enough O_2_	Breast cancer	36
ICD-boosting PDIT	Polymeric nanoparticle	HPPH and DOX	Enhance the population of TAAs and DCs recruitment by DOX inducing exposure of CRT and release of HMGB1	Colorectal cancer	37
	UCNP	RB and DOX	Enhance ICD by DOX inducing release of DAMPs	Breast cancer	133
	Boehmite	Ce6 and MLT	Express more DAMPs by MLT disrupting cell membrane via forming transmembrane pores	Breast cancer	134
	Liposome	Purpurin 18	Induce the exposed increase of CRT by the combination of PDT and PTT	Breast cancer	135
	Hollow gold/liposome	ICG/Hemoglobin	Boost ICD by ICG inducing synergistic PDT/PTT	Melanoma	43
	Nanoneedle	Aluminum phthalocyanine tetrasulfonate	Generate stronger ICD by hyperthermia and ROS generation	Cervical cancer	136
	Polymeric nanoparticle	TCPP	Amplify ICD by TCPP inducing endoplasmic reticulum stress	Breast cancer	137
ICD-boosting PDIT	Nanoaggregate	ICG	Induce powerful ICD based on enhanced PDT by discrete ICG concurrently alleviating ACQ and photobleaching	Breast cancer	138
	NCP nanoparticle	Pt and ICG	Tailor aggregation of ICG and integrate the complementarity of PDT/PTT/chemotherapy to magnify the ICD effect	Breast cancer	139
Adjuvant-promoted PDIT	MOF	H_2_TCPP, CpG and ACF	Release more cytokines by CpG activating TLR9 on the endosomal membrane	Liver cancer	33
	Polymeric nanoparticle	Ce6 and CpG	Promote DCs maturation by CpG activating TLR9	Melanoma	140
	Hydrogel	Ce6 and R837	Amplify the immunogenicity of TAAs by R837 activating TLR7 on the lysosome membrane	Breast cancer	45
ICB-combined PDIT	Nanoprobe	Phthalocyanine dye and anti-PD-1 antibody	Inhibit immune escape of tumors by anti-PD-1 antibody blocking PD-1/PD-L1 pathway	Breast cancer	141
	Polymeric nanoparticle	IR780 and anti-PD-L1 peptide	Enhance tumor infiltration of effector T cells by anti-PD-L1 peptide blocking PD-L1	Melanoma	142
	Polymeric micelle	PPa and siRNA	Improve immune response by siRNA inducing PD-L1 KD	Melanoma	39
	UCNP	Ce6, R837 and anti-CTLA4 antibody	Abrogate the activity of Tregs by anti-CTLA4 antibody blocking CTLA4	Colorectal cancer	9
	Polymeric nanoparticle	Zinc phthalocyanine and anti-CTLA4 antibody	Boost the activation of T cells by anti-CTLA4 antibody binding to CTLA4	Breast cancer	143
	Liposome	PpIX and NLG919	Increase Trp to enhance the activity of T cells by NLG919 inhibiting IDO	Breast cancer	47
	Polymeric nanoparticle	Ce6 and NLG919	Decrease Kyn to generate more CD8^+^ T cells by NLG919 interfering the activity of IDO	Colorectal cancer	144

## Abbreviations

PDT: photodynamic therapy
ROS: reactive oxygen species
ICD: immunogenic cell death
PDIT: photodynamic immunotherapy
TME: tumor microenvironment
ICIs: immune checkpoint inhibitors
O_2_: oxygen
PSs: photosensitizers
DAMPs: damage- associated molecular patterns
CRT: calreticulin
HMGB1: high mobility group box 1
ATP: adenosine triphosphate
DOX: doxorubicin
Ce6: chlorin e6
PpIX: protoporphyrin IX
R837: imiquimod
EPR: enhanced permeability and retention
APCs: antigen-presenting cells
DCs: dendritic cells
CAT: catalase
MnO_2_: manganese dioxide
H_2_O_2_: hydrogen peroxide
PFCs: perfluorocarbons
Hb: hemoglobin
PTT: photothermal therapy
CpG: cytosine-phosphate-guanine
TLRs: toll-like receptors
PD-1: programmed cell death protein 1
PD-L1: programmed death-ligand 1
CTLA4: cytotoxic T-lymphocyte-associated protein 4
IDO: indoleamine 2,3-dioxygenase
ICB: immune checkpoint blockade
MOFs: metal-organic frameworks
UCNPs: upconversion nanoparticles
NCPs: nanoscale coordination polymers
siRNA: small interfering RNA
PI3Kγ: phosphoinositide 3-kinase gamma
MDSCs: myeloid- derived suppressive cells
TIME: tumor immune microenvironment
GEM: gemcitabine
KD: knockdown
TPP: triphenylphosphonium
PLGA: poly(lactic-co-glycolic acid)
NPs: nanoparticles
FRT: ferritin
ZnF16Pc: zinc hexadecafuoro- phthalocyanine
TNF: tumor necrosis factor
IMT: imatinib
HA: hyaluronic acid
CD: cyclodextrin
AD: adamantine
BRD4: bromodomain and extraterminal protein 4
PPa: pyropheophorbidea
MMP-2: matrix metalloproteinase-2
MET: metformin
PDA: polydopamine
BP: black phosphorus
BPNV: BPQDs nanovesicle
2D: two- dimensional
SiO_2_: silica
bMSN: biodegradable mesoporous SiO_2_ NP
Fe_3_O_4_: ferriferrous oxide
M-MONs: magnetic mesoporous organosilicon NPs
TBP: 5,10,15,20-tetra(*p*-benzoato)porphyrin
EpCAM: epithelial cell adhesion molecule
MX: mitoxantrone
CAFs: cancer-associated fibroblasts
TAMs: tumor-associated macrophages
FAP: fibroblast activation protein
NRP-1: neuropilin-1
LINC: light-inducible nanocargo
CTLs: cytotoxic T lymphocytes
iPSs: induced pluripotent stem cells
CeO_2_: cerium oxide
ICG: indocyanine green
TiO_2_: titania
PEGDA: PEG double acrylate
OXA: oxaliplatin
Tregs: regulatory T cells
RB: rose bengal
MLT: melittin
ER: endoplasmic reticulum
ACQ: aggregation-caused-quenching
TAAs: tumor associated antigens
TABNs: thiol-activated bovine serum albumin NPs
HSP70: heat shock protein 70
MHC: major histocompatibility complex
Trp: tryptophan
Kyn: kynurenine
